# Supplementary figures and images for: Effects of vitamin B12 supplementation on neurodevelopment and growth in Nepalese Infants: A randomized controlled trial
Source: PLoS Med. 2020 Dec 1;17(12):e1003430. doi: 10.1371/journal.pmed.1003430 (PMC7707571; doi:10.1371/journal.pmed.1003430)

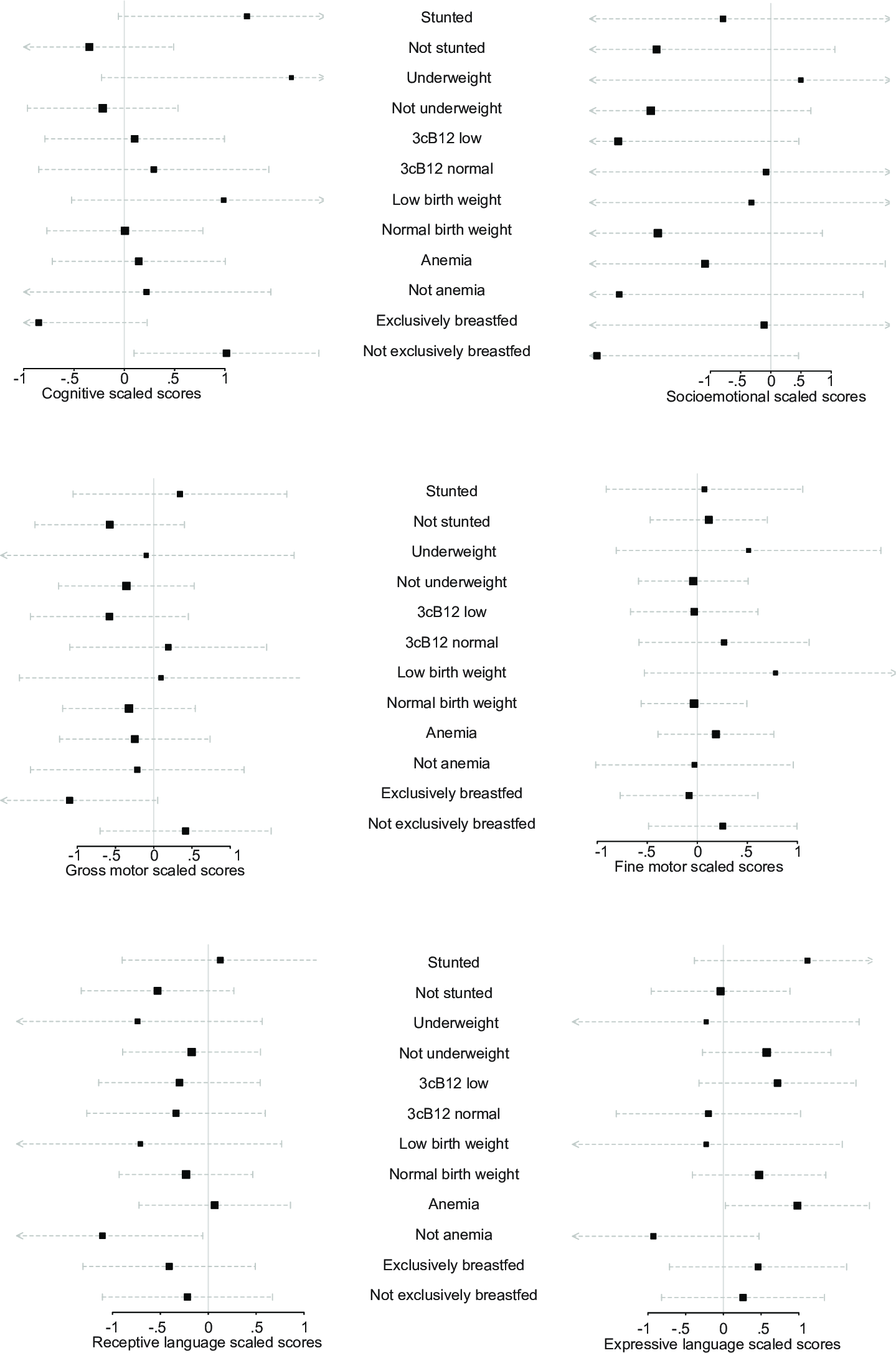

Supplement: S1 Fig — A point estimate to the left of the vertical line indicates a beneficial effect of vitamin B12. None of the subgroup specific effects were statistically significant. The effect estimates were calculated with multiple general linear models with the Gaussian distribution family and identity link function adjusting for length for age z-scores, maternal and paternal education, and age of the child at baseline. Stunting and underweight were defined as being <−2 length for age z-scores and weight for age z-scores, respectively. 3cB12: combined vitamin B12 status indicator as suggested by Fedosov and colleagues [28], low 3cB12 is <−0.5, low birth weight: birth weight <2,500 g, anemia: hemoglobin concentration <11 g/dL. (TIF) [file pmed.1003430.s001.tif]

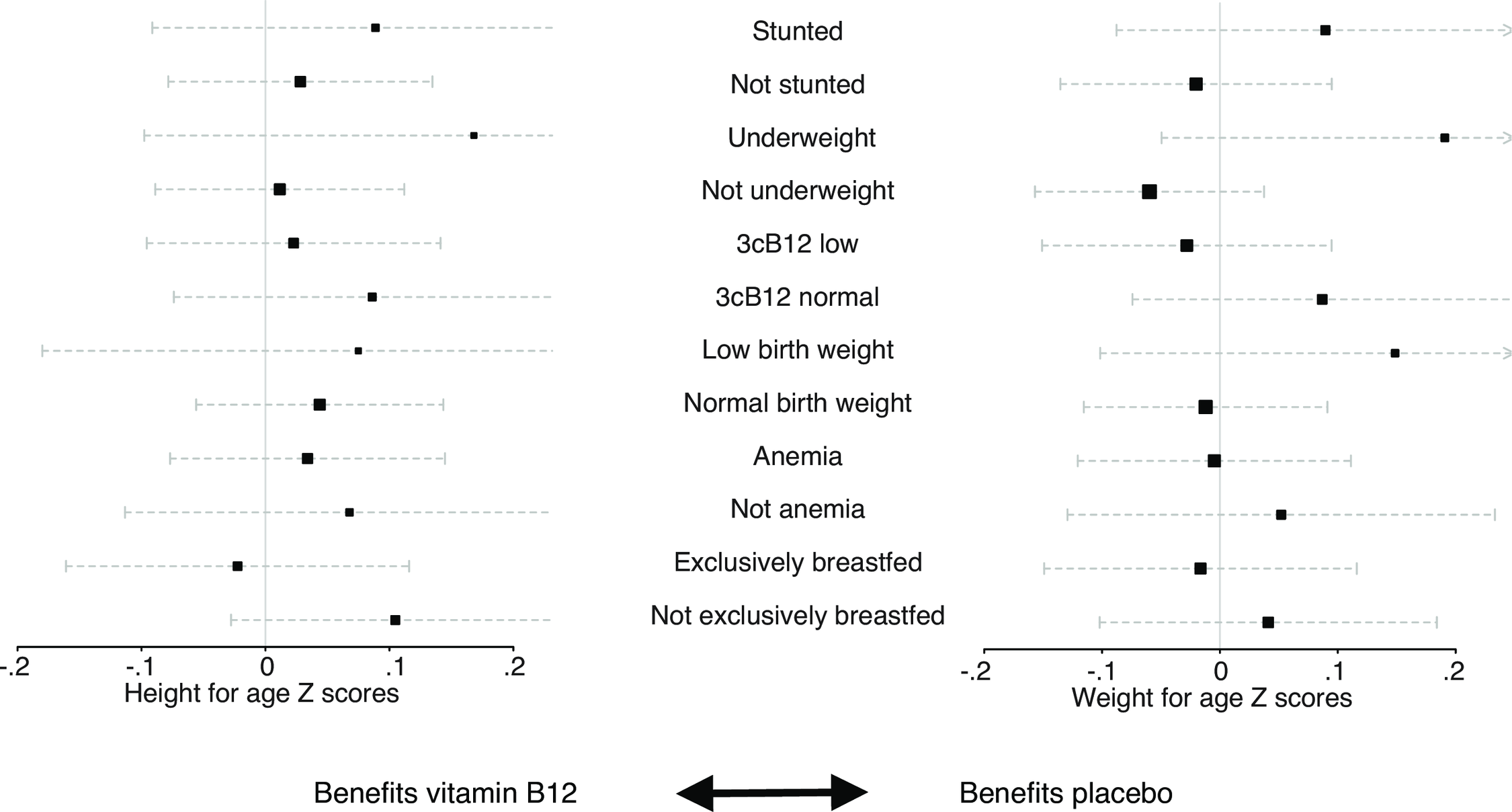

Supplement: S2 Fig — A point estimate to the left of the vertical line indicates a beneficial effect of vitamin B12. None of the subgroup specific estimates were statistically significant. The effect estimates were calculated with multiple general linear models with the Gaussian distribution family and identity link function adjusting for length for age z-scores, maternal and paternal education, and age of the child at baseline. Stunting and underweight were defined as being <−2 length for age z-scores and weight for age z-scores, respectively. 3cB12: combined vitamin B12 status indicator as suggested by Fedosov and colleagues [28], low 3cB12 is <−0.5, low birth weight: birth weight <2,500 g, anemia: hemoglobin concentration <11 g/dL. (TIF) [file pmed.1003430.s002.tif]
