# Supplementary material for: Effects of vitamin B12 supplementation on neurodevelopment and growth in Nepalese Infants: A randomized controlled trial
Source: PLoS Med. 2020 Dec 1;17(12):e1003430. doi: 10.1371/journal.pmed.1003430 (PMC7707571; doi:10.1371/journal.pmed.1003430)
Supplement: S1 Table — (DOCX) [file pmed.1003430.s003.docx]

**S 1 Table. Effects of daily vitamin B_12_ supplementation for one year starting in infancy on markers of vitamin B_12_ status**

|  |  | **Vitamin B_12_ group** | | |  | **Placebo group** | | |  | | **End-study ratio of**  **geometric means (GM)** | |  |
| --- | --- | --- | --- | --- | --- | --- | --- | --- | --- | --- | --- | --- | --- |
|  |  | ***n*** | **GM** |  |  | ***n*** | **GM** |  | |  | | **(95% CI)** | |
| **cobalamin**  **(pmol/L)** | |  | | |  |  |  |  | |  | |  | |
| All | baseline | 299 | 244.7 | (1.6) |  | 299 | 260.7 | (1.6) | |  | |  | |
|  | end-study | 277 | 316.3 | (1.7) |  | 286 | 290.1 | (1.6) | |  | | 0.92 (0.84 to 1.00) | |
| Deficient | baseline | 52 | 151.2 | (1.4) |  | 54 | 162.1 | (1.4) | |  | |  | |
|  | end-study | 46 | 266.5 | (1.6) |  | 53 | 212.9 | (1.6) | |  | | 0.80 (0.67 to 0.96) | |
| Low | baseline | 126 | 214.7 | (1.4) |  | 125 | 231.7 | (1.5) | |  | |  | |
|  | end-study | 118 | 279.9 | (1.6) |  | 119 | 283.1 | (1.6) | |  | | 1.01 (0.90 to 1.14) | |
| Adequate | baseline | 121 | 348.4 | (1.5) |  | 120 | 361.7 | (1.5) | |  | |  | |
|  | end-study | 113 | 387.8 | (1.7) |  | 115 | 337.5 | (1.6) | |  | | 0.87 (0.76 to 0.99) | |
| **total homocysteine (µmol/L)** | |  | | |  |  |  |  | |  | |  | |
| All | baseline | 300 | 10.7 | (1.5) |  | 299 | 11.0 | (1.6) | |  | |  | |
|  | end-study | 280 | 6.6 | (1.3) |  | 287 | 8.2 | (1.4) | |  | | 1.23 (1.17 to 1.30) | |
| Deficient | baseline | 52 | 18.2 | (1.5) |  | 54 | 19.8 | (1.5) | |  | |  | |
|  | end-study | 46 | 7.9 | (1.4) |  | 52 | 11.1 | (1.5) | |  | | 1.41 (1.21 to 1.64) | |
| Low | baseline | 126 | 11.7 | (1.4) |  | 125 | 11.9 | (1.4) | |  | |  | |
|  | end-study | 118 | 6.8 | (1.3) |  | 119 | 8.6 | (1.4) | |  | | 1.26 (1.18 to 1.36) | |
| Adequate | baseline | 122 | 7.7 | (1.3) |  | 120 | 8.0 | (1.3) | |  | |  | |
|  | end-study | 116 | 6.0 | (1.3) |  | 116 | 6.9 | (1.3) | |  | | 1.15 (1.07 to 1.23) | |
| **methylmalonic acid (µmol/L)** | |  | | |  |  |  |  | |  | |  | |
| All | baseline | 300 | 0.5 | (2.2) |  | 299 | 0.5 | (2.3) | |  | |  | |
|  | end-study | 280 | 0.3 | (1.9) |  | 287 | 0.4 | (2.1) | |  | | 1.30 (1.15 to 1.46) | |
| Deficient | baseline | 52 | 1.4 | (1.8) |  | 54 | 1.5 | (1.8) | |  | |  | |
|  | end-study | 46 | 0.5 | (2.1) |  | 52 | 0.8 | (2.1) | |  | | 1.60 (1.20 to 2.14) | |
| Low | baseline | 126 | 0.5 | (1.6) |  | 125 | 0.6 | (1.7) | |  | |  | |
|  | end-study | 118 | 0.4 | (1.8) |  | 119 | 0.5 | (1.9) | |  | | 1.47 (1.25 to 1.72) | |
| Adequate | baseline | 122 | 0.2 | (1.6) |  | 120 | 0.3 | (1.6) | |  | |  | |
|  | end-study | 116 | 0.3 | (1.8) |  | 116 | 0.3 | (1.9) | |  | | 1.07 (0.92 to 1.25) | |
|  |  |  |  |  |  |  |  |  | |  | |  | |

The mean differences and the corresponding 95 % CI were calculated on the log-transformed concentrations using the Student’s t-test assuming equal variances. Deficient, Low and Adequate defined by the combined vitamin B_12_ status indicator (3cB12) as suggested by Fedosov et. Al [28]. *n*: Number analyzed, GM: geometric means, numbers in parentheses are the geometric standard deviation factor, which is the exponentiated SD of the logarithmic mean. The end-study ratios are the ratios between the GM in the placebo group over the vitamin B_12_ group. When the confidence interval includes 1, the ratio is not statistically significant. Thus, all but two of the ratios at end-study were statistically significant (p<0.05).
