# Supplementary material for: Effects of vitamin B12 supplementation on neurodevelopment and growth in Nepalese Infants: A randomized controlled trial
Source: PLoS Med. 2020 Dec 1;17(12):e1003430. doi: 10.1371/journal.pmed.1003430 (PMC7707571; doi:10.1371/journal.pmed.1003430)
Supplement: S2 Table — (DOCX) [file pmed.1003430.s004.docx]

**S 2 Table. Compliance of vitamin B_12_ supplementation among Nepalese infants participating in clinical trial on the effect of vitamin B_12_ supplementation on growth, development, and hemoglobin concentration**

|  | **Vitamin B_12_ group** | | **Placebo group** | | |
| --- | --- | --- | --- | --- | --- |
| Information on whether supplements was given or not | n | % | n | % |  |
|  | 98751 |  | 97422 |  |  |
| All paste | 80697 | 81.7 % | 78022 | 80.1 % |  |
| No paste | 5703 | 5.8 % | 5830 | 6.0 % |  |
| Half | 8132 | 8.2 % | 8848 | 9.1 % |  |
| One third | 1674 | 1.7 % | 1513 | 1.6 % |  |
| One forth | 2545 | 2.6 % | 3209 | 3.3 % |  |
|  |  |  |  |  |  |
| Caregiver did not remember whether supplements was not given or not | 4640 | 4.7 % | 5750 | 5.9 % |  |
|  |  |  |  |  |  |
| Why not given or why a reduced amount was given |  |  |  |  |  |
|  | 18054 | 18.3 % | 19400 | 19.9 % |  |
| Illness | 3569 | 3.6 % | 3791 | 3.9 % |  |
| Difficult to feed | 4007 | 4.1 % | 4129 | 4.2 % |  |
| Did not like | 8061 | 8.2 % | 9127 | 9.4 % |  |
| Forgot to give | 1373 | 1.4 % | 1410 | 1.4 % |  |
| Travelling | 503 | 0.5 % | 403 | 0.4 % |  |
| No reason given | 541 | 0.5 % | 540 | 0.6 % |  |
