# Supplementary material for: Effects of vitamin B12 supplementation on neurodevelopment and growth in Nepalese Infants: A randomized controlled trial
Source: PLoS Med. 2020 Dec 1;17(12):e1003430. doi: 10.1371/journal.pmed.1003430 (PMC7707571; doi:10.1371/journal.pmed.1003430)
Supplement: S3 Table — (DOCX) [file pmed.1003430.s005.docx]

**S 3 Table. Adverse effects of vitamin B_12_ supplementation among Nepalese infants participating in clinical trial on the effect of vitamin B_12_ supplementation on growth, development, and hemoglobin concentration**

|  | **Vitamin B_12_ group** | | **Placebo group** | |
| --- | --- | --- | --- | --- |
| **Hospitalizations**  Number of children | 20 |  | 16 |  |
| Number of events | 26 |  | 17 |  |
| Child with one events | 15 |  | 15 |  |
| Child with two events | 4 |  | 1 |  |
| Child with three events | 1 |  | 0 |  |
|  |  |  |  |  |
| **Specific diagnosis of each event** |  |  |  |  |
| Urinary tract infections | 3 |  | 0 |  |
| Lower respiratory infections | 8 |  | 10 |  |
| Seizure related illness | 7 |  | 2 |  |
| Constipation | 1 |  | 1 |  |
| Other (surgical, diarrhea, sepsis, etc.) | 7 |  | 4 |  |
|  |  |  |  |  |
| **Day-wise information on adverse events** |  |  |  |  |
| **Days with available information** | 99,214 |  | 97,991 |  |
| Vomiting |  |  |  |  |
| Yes | 141 | 0.1 % | 134 | 0.1 % |
| No | 93,049 | 93.8 % | 91,563 | 93.4 % |
| No information from this day | 6024 | 6.1 % | 6294 | 6.4 % |
|  |  |  |  |  |
| Diarrhea | 99,216 |  | 97,996 |  |
| Yes | 5033 | 5.1 % | 5076 | 5.2 % |
| No | 93,703 | 94.4 % | 92,339 | 94.2 % |
| No information from this day | 480 | 0.5 % | 581 | 0.6 % |
|  |  |  |  |  |
| Visits to health centers | 99,216 |  | 97,996 |  |
| yes | 2180 | 2.2 % | 1996 | 2.0 % |
| no | 96,554 | 97.3 % | 95,416 | 97.4 % |
| Do not remember | 482 | 0.5 % | 584 | 0.6 % |
