# Supplementary material for: Effects of vitamin B12 supplementation on neurodevelopment and growth in Nepalese Infants: A randomized controlled trial
Source: PLoS Med. 2020 Dec 1;17(12):e1003430. doi: 10.1371/journal.pmed.1003430 (PMC7707571; doi:10.1371/journal.pmed.1003430)
Supplement: S2 Text — October 16, 2016. (DOCX) [file pmed.1003430.s007.docx]

**The effect of Vitamin B12 supplementation on Growth and Development in Nepali Infants**

Main protocol version 2.1

October 16, 2016

**CIH:** Centre for International Health, University of Bergen, Norway

**TU:** Department of Child Health, Tribhuvan University, Nepal

**IHT:** Department of Research, Innlandet Hospital Trust, Norway

**UniHealth:** UniHealth, UniResearch Bergen, Norway

**Principal Investigator:**

Tor A Strand MD/PhD (IHT/CiH)

Professor

Innlandet Hosptial Trust

**Other Investigators:**

Ram K Chandyo MD/PhD (TU)

Mari Hysing PsyD/PhD (UniHealth)

Ingrid Kvestad PsyD/PhD (UniHealth)

Laxman Shrestha MD (co-PI) (TU)

Merina Shrestha MD (TU)

Prakash S Shrestha MD/PhD (co-PI) (TU)

Manjeswori Ulak MD (TU)

| **Lay Abstract** |
| --- |
| **Rationale:** Globally, vitamin B12 deficiency is one of the most common micronutrient deficiencies. The only relevant source of Vitamin B12 is animal-source foods. Vitamin B12 is crucial for normal cell division and is necessary for brain growth as well as for the maintenance of its normal function. Deficiency is also associated with impaired growth. In a previous study, we demonstrated that vitamin B12 administration over a period of six months enhanced growth, and scores on a neuro-developmental test in young Indian children. However, the overall effect was small and, for the developmental scores significant only in those that were malnourished at the start of the study.  Our findings need to be verified in trials targeting younger, malnourished children and with longer supplementation time.  **Hypothesis***:* This proposed study will test three hypotheses; to measure to what extent 2 µg of vitamin B12 administration for one year to marginally stunted children improves; 1) growth, 2) neurodevelopment, and 3) hemoglobin concentration.  **Study design:** Randomized placebo-controlled trial. Half of the children will receive a paste containing vitamin B12, the other half the same paste but without vitamin B12.  **Study participants and site:** 600 malnourished infants in Bhaktapur municipality in Nepal. In this population we have demonstrated that vitamin B12 deficiency and poor growth is common in early childhood.  **Intervention:** Daily administration of a paste containing vitamin B12 or placebo for 12 months  **Data:** The main outcomes of this study are scores on developmental assessments tools and growth measured every month for 12 months. We will also attempt to follow these children beyond the time of supplementation, i.e. beyond the period of this project to measure to what extent any neurodevelopmental changes persist after end of supplementation.  **Goal:** A substantial proportion of children in many low and middle-income countries suffer from poor growth, which is associated with poor health and poor brain development. Our goal is to identify modifiable limitations for growth and brain functioning in young children in poor settings. If vitamin B12 is such a limitation, increasing the intake in susceptible populations will be the next step. The approach will depend on the setting and what is acceptable in the target population. In Nepal, this can be done by promoting available inexpensive animal-source foods such as animal milk and eggs. In other places, routine supplementation, food fortification or even regular injections may be the most feasible approach. The body has the ability to store vitamin B12 for a long time, i.e. supplementation for limited time periods may have a long –term impact on vitamin B12 status, growth and cognitive development in many children worldwide. Thus, there are several non-expensive ways of improving vitamin B12 status in young children in poor countries if this is necessary. A recent review in the Lancet estimated that there may be more than 200 million children worldwide who fail to reach their developmental potential, ensuring adequate vitamin B12 status to many of these may help bring this too high number down. |

| **Scientific Abstract** |
| --- |

**Scientific basis:** Globally, vitamin B12 deficiency is one of the most common micronutrient deficiencies. The only relevant source of vitamin B12 is animal-source foods. Vitamin B12 is crucial for normal cell division and differentiation and is necessary for the development and initial myelination of the central nervous system as well as for the maintenance of its normal function. Deficiency is also associated with impaired infant and child growth.

In a previous clinical trial, we demonstrated that vitamin B12 administration over a period of six months enhanced growth and neurodevelopment in young Indian children. The overall effect on growth was significant but small. We saw an effect on both ponderal (weight for age z scores) and linear (height for age z scores) growth. However, the overall effects were driven by the effects in the subgroups of children who were wasted, underweight or stunted at baseline, and no effect in the children who were not malnourished at baseline. This effect modification was significant for all three (stunting, wasting, and underweight) baseline variables. Similarly, the effect of the intervention on neurodevelopmental scores was also strongest in the subgroup of children that were stunted.

We have for the last 15 years undertaken studies on dietary intake and status in women and children in Bhaktapur, Nepal. In this site, vitamin B12 deficiency is very common. The objective of the proposed study is to measure to what extent administration of 2 µg of vitamin B12 to stunted children from the last half of infancy and for 12 months affect neurodevelopment, growth and hemoglobin concentration.

***Hypothesis:*** Daily supplementation of 2 µg of vitamin B12 in young Nepali children for 12 months improves neurodevelopment, growth and hemoglobin concentration.

**Study design:** Individually randomized placebo controlled, double blind trial. Children will be identified in the community and children will be randomized to daily receive a paste containing vitamin B12 or a placebo paste. The paste will be delivered by trained field workers every day and by the caregivers on Saturdays and public holidays.

**Study participants and site:** 600 marginally stunted children aged 6 to 11 months in Bhaktapur municipality and surrounding areas.

**Intervention:** Daily administration of a paste containing vitamin B12 or placebo for 12 months.

**Comparator:** Placebo, identical to the vitamin B12 supplements.

**Data:** Primary outcomes: (i) neurodevelopmental scores measured by Bayley Scales of Infant and Toddler Development 3^rd^ edition and the Ages and Stages Questionnaire 3^rd^ edition after 6 and 12 months of supplementation (ii) growth measured by change in height for age, weight for age and weight for height z-score from study start to end study and growth velocity z scores during the six first and six last months of supplementation (iii) hemoglobin concentration after 12 months of supplementation. Secondary: (i) cognitive development in children measured between 2 and 6 years after enrollment, (ii) linear and ponderal growth measured 2 to 6 years after enrollment, (iii) hemoglobin concentration measured 2 and 3 years after enrollment, (iv) leucocyte telomere length as a marker of biological age, (v) vagal tone (heart rate variability), and (vi) distractibility (eye tracking measurements). All secondary outcomes require additional funding.

**Relevance for programs and public health:** Improved learning ability and growth in young malnourished children. If the supplementation is effective this will have consequences for dietary recommendation to malnourished children worldwide. In contrast to most other relevant nutritional interventions, vitamin B12 is inexpensive and our body has the ability to store vitamin B12, up to years. Thus, improving the status of this nutrient for a limited time period may have impact on learning and productivity beyond the time of administration and help to lift poor children out of the vicious cycle of poverty and malnutrition.

| Hypothesis(es) and Aims |
| --- |
| A. Hypothesis(es) to be Tested |
| 1. Daily administration of 2 µG of vitamin B12 to 6 to 11 months old children for 12 months improves neurodevelopment in early childhood. 2. Daily administration of 2 µG of vitamin B12 to 6 to 11 months old children for 12 months improves linear and ponderal growth. 3. Daily administration of 2 µG of vitamin B12 to 6 to 11 months old children for 12 months improves hemoglobin concentration. |
| B. Specific Aim(s) of the Project |

In a population of infants 6 to 11 months of age and where vitamin B12 deficiency and malnutrition is prevalent:

1. measure to what extent administration of 2 µg of vitamin B12 for one year improves developmental scores measured by the Bayley Scales of Infant and Toddler Development 3^rd^ edition (BSID-III) and the Ages and Stages Questionnaire 3^rd^ edition (ASQ-3),
2. measure to what extent administration of 2 µg of vitamin B12 improves length for age z-scores after 1 year and length growth velocity Z scores for the initial 6 and last 6 months of supplementation
3. measure to what extent administration of 2 µg of vitamin B12 improves weight for age and weight for length z-scores after 1 year and growth velocity Z scores for the initial 6 and last 6 months of supplementation,
4. measure to what extent administration of 2 µg of vitamin B12 for 12 months improves hemoglobin concentration.
5. identify subgroups for the above-mentioned outcomes that benefit from vitamin B12 administration.
6. measure to what extent vitamin B12 administration alters plasma vitamin B12, plasma methyl malonic acid and plasma total homocysteine concentration.
7. improve the competence in developmental and cognitive assessment in Nepal .
8. in multiple regression analyses, identify predictors for early child development scores using variables reflecting socioeconomic status, early stimulation opportunities, morbidity, growth, and micronutrient status.
9. Measure the extent to which the intervention has an impact on neurological function measured by heart rate variability or eye tracking
10. Measure the extent to which the intervention or any other of the above-mentioned exposures are associated with leucocyte telomere length.

| **Background and Significance** |
| --- |

**Vitamin B12 deficiency:** Micronutrient deficiencies represent a major challenge to child health in many low and middle-income countries (LMIC) and may be associated with suboptimal cognitive function and poor growth ([1](#_ENREF_1)). Studies in children and adults have shown that subtle vitamin B12 deficiency is common ([2](#_ENREF_2), [3](#_ENREF_3)). This is not surprising as the main source of vitamin B12 is animal source foods, which are expensive and for cultural and religious reasons often not eaten at all. Vitamin B12 is required for the folate-dependent enzyme, methionine synthase, which is necessary for the synthesis of methionine from homocysteine (tHcy). Methionine in its activated form, S-adenosylmethionine, is the major methyl group donor used in human methylation reactions, including methylation of DNA and RNA. Impaired vitamin B12 status has consequences for DNA and protein synthesis and therefore cell growth and differentiation ([4](#_ENREF_4)). Poor children who are malnourished might also have poor vitamin B12 status, which again may contribute to their impaired growth. Stunted growth is associated with several poor health outcomes including increased risk of infections and adverse brain development.

**Vitamin B12 and cognitive development:** In observational studies, having low levels of vitamin B12 is related to cognitive decline and dementia in adults and elderly ([5](#_ENREF_5)). Several studies have also shown that vitamin B12 deficiency is associated with cognitive deficits even at concentrations above the traditional cutoffs for deficiency ([6](#_ENREF_6)). Although fewer epidemiological studies, the association between cognitive functioning and vitamin B12 status has also been described in children. Neonatal vitamin B12 deficiency causes failure to thrive and neurologic manifestations, which may become irreversible ([7](#_ENREF_7)). A study in the Netherlands showed that infants raised on a macrobiotic diet (vegetarians) had delayed gross motor and speech and language development on a standardized checklist compared to infants on an omnivore diet (non-vegetarian) ([8](#_ENREF_8)). In adolescence, the children raised on a microbiotic diet for the six first years of life had poorer performance on cognitive tests independently of current vitamin B12 status compared to adolescents who were raised on an omnivorous diet ([9](#_ENREF_9)).

In a cohort of young Nepalese children, intake of meat and other animal products, which are good sources of vitamin B12 was positively associated with cognitive performance ([10](#_ENREF_10)). In a cohort study in North India we demonstrated that children with poor vitamin B12 status measured by low plasma vitamin B12, or elevated methylmalonic acid (MMA) or tHcy, had lower Mental development scores on the Bayley Scales of Infant and Toddler Development 2nd. edition (BSID-II) than those who did not have signs of vitamin B12 deficiency ([11](#_ENREF_11)). These associations remained strong and significant even after adjusting for potential confounders such as socioeconomic status, maternal and paternal education, growth status, age, and sex and are supported by a recently completed randomized, placebo-controlled trial we conducted in Indian children 6 to 30 months of age (see “Supportive Preliminary Data**”)** ([12](#_ENREF_12)).

**Brain development:** The first two years of life is the most important period for brain growth and development of the nervous system ([13](#_ENREF_13)). The brain develops rapidly through neurogenesis, axonal and dendritic growth, synaptogenesis, myelination, and gliogenesis. These events happen at different times and build on each other ([13](#_ENREF_13)). Small impairments during critical periods can have serious consequences for the brain’s structural and functional capacity with the possibility of persistence into adult life. It is the functions and areas under development that are most sensitive to negative influences, and provide the most specific outcomes in assessments ([14](#_ENREF_14)) ([15](#_ENREF_15)). However, the functions under development at the time of negative exposure are not necessary the salient function at the time of assessment ([16](#_ENREF_16)). Consequently, to fully understand findings in developmental assessments, we must consider the developmental timing of an exposure, in this case vitamin B12 supplementation (or lack thereof), as well as the timing of the assessment ([14](#_ENREF_14)).

The previously mentioned Dutch study indicated consequences of early vitamin B12 deficiency on cognitive performance in adolescence, independently current vitamin B12 status ([17](#_ENREF_17)). In early childhood, children with poor B12 status had significant slower gross motor and speech and language development. These results indicate early influences to the participants developing brain, however with different developmental expressions depending on the timing of assessment ([17](#_ENREF_17)). A causal relationship between vitamin B12 status and motor development was recently demonstrated in a randomized clinical trial: Otherwise healthy Norwegian infants who visited an outpatient clinic for feeding problems and who had symptoms of mild vitamin B12 deficiency (plasma tHcy > 6.5 µmol/L) were randomized to receive an intramuscular injection of vitamin B12 or placebo (28). One month after the intervention, children who received vitamin B12 had substantially improved their motor function and experienced less regurgitation. The study did not, however, measure the effect of the intervention on other aspects of early child development.

**Why may vitamin B12 be important for the developing brain:** Poor vitamin B12 status leads to a deﬁciency of S-adenosylmethionine, which again causes deficient methylation reactions in the central nervous system. This can impair the methylation of myelin basic protein in the central as well as peripheral nervous system ([6](#_ENREF_6)). The production of myelin is a key component of brain development from gestation, throughout childhood and well into middle age. The myelination of the brain is of importance for multiple brain systems and is highly related to neurodevelopment and subsequent cognitive functioning. Furthermore, vitamin B12 is important for cell division and maturation, which again is crucial for adequate development of the central nervous system. Vitamin B12 also contributes by serving as a cofactor in numerous catalytic reactions in the human body, which are required for neurotransmitter synthesis and functioning. Vitamin B12 deficiency may cause pernicious anemia with similar effects on cognitive development and functioning as anemia caused by iron deficiency ([18](#_ENREF_18)). Vitamin B12 deficiency can also result in neuropathy through degeneration of nerve fibers and irreversible brain damage ([19](#_ENREF_19)) ([6](#_ENREF_6))

**Micronutrients and cognitive development:** A recently published meta-analysis showed that supplementation with several micronutrients to children can improve cognitive development ([20](#_ENREF_20)). This meta-analysis included several studies but only three studies in children less than 5 years of age, which is the most critical time of brain development. Of these three studies, only one study included vitamin B12 as part of the multi micronutrient formulation ([21](#_ENREF_21)). This study showed that children, who were given fortified food that included vitamin B12, had significantly higher motor development scores compared to those who weren’t.

**Other factors that are important for cognitive development:** There is sound evidence that deficient care and inadequate stimulation are key risk factors for adverse neurodevelopment in children. Poverty, improper care and malnutrition are associated and in the same pathway leading to poor development. Furthermore, the interaction between caregiver and child may be affected by the behavioural consequences of a child`s nutritional status and thus result in improper development.

Negative influences during early childhood can have important consequences for brain functioning for the rest of a person’s life. Vitamin B12 is crucial for the developing central nervous system and even mild deficiency is associated with impaired neurodevelopment. Many young infants suffer from subtle vitamin B12 deficiency. Establishing a causal link between mild deficiency and neurodevelopment has to be done in a randomized, placebo controlled trial that provide adequate amounts of vitamin B12 for a sufficiently long period.

If we are able to demonstrate a causal relationship between vitamin B12 and cognitive functioning in malnourished children in low-income countries, this may contribute to national nutrition recommendations and the recommended composition of complementary foods. Other trials have measured the impact of several micronutrients on growth and neurodevelopment in poor populations; many of these did not include vitamin B12. Most of these also failed to show an effect on growth or neurodevelopment. In fact, the only study on multi-micronutrient administration in children under the age of 5 years that had effect on neurodevelopment included vitamin B12 in their multivitamin formulation. Demonstrating that vitamin B12 is important for neurodevelopment and/or growth can ensure that vitamin B12 is included in micronutrient formulations that are recommended or available to vulnerable populations. This is indeed not always the case. The target population in Bhaktapur, Nepal is relatively food secure, they have good access to health facilities and are well off compared to other children in South Asia. Despite this, the rates of stunting, wasting and vitamin B12 deficiency are high. If vitamin B12 is an important growth and development –limiting factor, supplementation of this single nutrient has the potential to improve growth and development in many children. In contrast to many other micronutrients, vitamin B12 can be stored in the body and the vitamin B12 status can be improved by a bolus dose such as for vitamin A.

**Telomeres and vitamins:** Telomeres are the caps at the end of each strand of the DNA that protects our chromosomes. Telomere length is an indication of remaining cell cycles of a particular cell line in the body, and shorter telomeres in the individual are a sign of shortened life span. Stress may accelerate telomere shortening and the length of telomeres is often considered to be a biomarker of chronic stress.

In a previous study in Nepal, we measured the relative telomere length in leukocytes in 66 infants using frozen blood. In this study, we found that, in addition to decrease by age, the telomere length was negatively associated with maternal vitamin B12 levels measured by plasma methylmalonic acid (MMA). Thus poor vitamin B12 status in mothers reflected in high plasma MMA concentrations was associated with shorter telomeres in the offspring during infancy. In low and middle-income settings poverty is linked with a range of stressors in early childhood that can confound this observed and unpublished association.

| **Supportive Preliminary Data** |
| --- |

**Vitamin B12 deficiency:** We have assessed the statues and intake of several nutrients in Nepal and India for more than 15 years. We have found that vitamin B12 is one of the most commonly occurring deficiency and that it is linked with poor growth and poor cognitive development independently of other nutritional factors and other background variables. In the target population we have also measured nutrient deficiencies in women of reproductive age. These women suffer from the same nutritional deficiencies as young children, including vitamin B12 deficiency. The vitamin B12 results are summarized in the table below. It should be noted that the cut offs for defining vitamin B12 deficiency in young children are not well defined. However, our findings show that the mean concentration of vitamin B12 is much lower, and that the mean concentrations of MMA and tHcy are higher in Nepal and India than in most populations in Europe and in the US. The functional markers MMA and tHcy are elevated when there is B12 deficiency, and are very sensitive to even mild deficiency.

Table. Studies on vitamin B12 status in Nepal and India

-Very low B12 < 148 pmol/L, low B12 < 222 pmol/L, high tHcy > 10 µmol/L, high MMA > 0.47 µmol/L (child), high MMA > 0.28 µmol/L (adult)

-ALRI: Acute Lower Respiratory Infections

**Vitamin B12 and neurodevelopment.** In the cohort of children in India 12 to 18 months old at enrolment, we also measured cognitive performance at two occasions 4 months apart ([11](#_ENREF_11)). Both vitamin B12 and folate status were associated with poor developmental scores (BSID-II). From 2010 we undertook a randomized, placebo controlled trial funded by the Thrasher Research fund where we provided 2 µG of placebo, vitamin B12, folic acid, or both vitamins for six months to children aged 6-30 months at enrolment. The main objective of this trial was to measure the effect of vitamin B12 and/or folic acid on the incidence of infections. Neither of the nutrients led to a reduction in the incidence of diarrhea or respiratory tract infections. A secondary outcome of this trial was neurodevelopment measured by the developmental screening tool ASQ-3. We found that children who received both vitamin B12 and folic acid had approximately 15 points higher ASQ-3 scores than children who received placebo (*P* = 0.051), and these children had lower odds of being in the lower quartile of the ASQ-3 scores (Odds ratio 0.53, 95% CI: 0.29 - 0.99, *P* = 0.048). There was no significant effect of vitamin B12 or folic acid alone. Stunted children, and children with elevated total homocysteine had significantly reduced odds of receiving scores in the lower quartile of the ASQ-3 scores [OR 0.26, 95% CI 0.09-0.78, *P* = 0.016) and (OR 0.38, 95% CI 0.16-0.92, *P* = 0.032) respectively]. Children who received vitamin B12 and folic acid supplementation before the age of 2 years achieved 20 ASQ-3 points higher than children who received placebo (P=0.01). All effects were most apparent in the domains of gross motor and problem solving skills.

**Growth.** In Bhaktapur Nepal, as in many other poor settings, the prevalence of childhood stunting increases by age after the 4^th^ month of life. In fact, data from our ongoing MAL-ED (mal-ed.fnih.org) project demonstrates that before the 4^th^ months of life less than 10% of the children are at risk for stunting (<-1 zscores length for age). When the cohort reached 2 years of age, approximately half of the children were stunted. These figures are supported by other studies in the same population during the last 15 years. Thus, the period between late infancy and their 2^nd^ birthday is important for intervening against poor growth.

**Vitamin B12 and growth.** In the completed vitamin B12 and folic acid trial in New Delhi, growth was a predefined secondary outcome. Compared to placebo, administration of vitamin B12 in combination with folic acid increased the mean weight and length by 120 g (95% CI: 3-210 g) and 0.29 cm (CI: 0- 58), respectively. When restricting the analyses to children that were stunted, wasted or underweight at time of randomization, the effects on all indices of growth (including change in Z scores height and weight for age) were stronger and statistically significant following vitamin B12 supplementation. We saw an effect of vitamin B12 both in children who were given folic acid as well as in those who were not, i.e. the effect of vitamin B12 was independent of folic acid supplementation. Furthermore, the interactions between B12 administration and stunting, wasting or underweight on growth were statistically significant for all relevant growth outcomes (Strand, submitted).

**Vitamin B12 and anemia.** In Nepalese and Indian children we found that approximately half of the children were anemic and that vitamin B12 status was associated with hemoglobin concentration (Ulak, in review 2013, Kumar, in press, 2013). Anemia is also linked to poor cognitive outcomes in young children in low and middle-income countries. Poor vitamin B12 status could explain part of this association.

**Why is demonstrating a causal effect of vitamin B12 administration on neurodevelopment important.** In the above mentioned cohort of Indian children, we demonstrated that vitamin B12 deficiency is common and associated with lower scores on the BSID-II mental development index ([11](#_ENREF_11)). Vitamin B12 status was also the main predictor for elevated tHcy, elevated tHcy is a sign of functional vitamin B12 deficiency ([22](#_ENREF_22)) ([3](#_ENREF_3)). In our samples of children from Nepal and India, tHcy starts to rise when vitamin B12 is lower than 250 – 300 pmol/L, which is higher than the traditional cut-off used for defining vitamin B12 deficiency. This indicates that subtle B12 deficiency is very common. If there is a causal relationship between poor vitamin B12 intake and neurodevelopment, efforts should be made to improve vitamin B12 status in children and possibly also in pregnant women. However, before such recommendations are made, the above-mentioned results from observational studies and one clinical trial need to be verified in randomized, placebo-controlled trials designed for this purpose. Initially this should be done under circumstances that ensure high internal validity of the study such as in the proposed clinical trials. In this study we will do so by focusing on a relative narrow age range, by ensuring high compliance and by recruiting vulnerable, i.e. stunted, children.

The clinical trial on folic acid and vitamin B12 in New Delhi was designed to measure an impact of folic acid and/or vitamin B12 administration on the incidence of infections. Growth and neurodevelopment were secondary outcomes. A study designed to measure the effect on growth and development of the central nervous system should provide the intervention for longer time than in a study that is designed to measure an effect on infections. Furthermore, the age range should be narrower and focus on children less than 2 years of age, and more comprehensive developmental assessment tools should be utilized in addition to or instead of ASQ-3.

This proposed project is designed to measure the effect of vitamin B12 on growth, neurodevelopment and hemoglobin concentration. We will undertake the study within the critical window for brain development. We will start after other foods (in addition to breast milk) are introduced to the child and end before the child reaches 24 months of age. This is also a population where stunting is common and, in contrast to the cohort that we have studied in North India, folate deficiency is virtually non-existent. The duration of vitamin B12 administration will be twice as long as the duration of supplementation in the study in India and the children are younger and thus with a greater potential for catch-up growth.

Thus, we believe that the proposed study is well designed for measuring the effect of vitamin B12 on cognitive outcomes, growth, and on hemoglobin concentration. We will do this in a population where we have more than 15 years of working experience

| **Experimental Design and Methodology** |
| --- |

**Design:** Randomized, double blind placebo controlled field trial. Enrolled children will receive placebo or vitamin B12 daily for 12 months.

The impact of supplementation with vitamin B12 will be measured using a randomized double-blind placebo-controlled study design. Placebo or vitamin B12 supplementation will be maintained at home for 12 months. The supplements will be provided by trained field workers daily and by caregivers on Saturdays and public holidays. If regurgitation/vomiting occur within 15 minutes of supplementation, extra dose will be given.

Study site. The study site is located in Bhaktapur municipality, which is 15 km east of the capital city Kathmandu. Bhaktapur is an ancient city famous for its traditional temples and buildings. It is listed under UNESCO world heritage and one of the main tourist attractions in Nepal. Bhaktapur is a homogenous community consisting mostly of practicing mixed Hindus and Buddhists. It is a peri-urban agricultural based community located 1400 m above sea level with a population predominated by the Newar ethnic group. The senior most member of the family is usually considered as the head of household. In the Bhaktapur municipality, domestic migrant workers from diverse ethnic groups work in several carpet factories. The local climate is humid subtropical with wet and hot season (monsoon) from May to August and dry and cool season from October to March. The annual average rainfall is 78.3 mm and the temperature ranges from -2 to 35 ^o^C. Majority of the residents in Bhaktapur consume foods grown in the community. Rice is the staple food. The eating pattern varies with season, workload in the field and availability of foods. A variety of local green leafy vegetables are widely consumed mainly in the winter and spring season.

**Criteria for inclusion of children.** Children will be enrolled if they fulfill the following inclusion criteria:

*Age 6 to 11 months

* Length for age Z scores <-1 (marginally stunted)

* Availability of informed verbal consent

* Plan to reside in the area for the next 12 months

**Criteria for exclusion of children.**

- Severe systemic illness requiring hospitalization

- Severe malnutrition, i.e. weight for height < -3 z of the WHO standard for this age group. For ethical reasons these children require micronutrient supplementation and adequate medical care.

- Lack of consent

- Taking B vitamin supplements that include vitamin B12.

- Severe anemia (Hb < 7 g/dL). This would be a temporary exclusion and the children will be enrolled if they are successfully treated.

- Ongoing acute infection with fever or infection that requires medical treatment. This would be a temporary exclusion and the children will be enrolled after recovery.

**Random allocation to intervention groups and blinding procedures.** Eligible children will be allocated by block randomization (block sizes of 8) to one of the intervention groups outlined in the figure. supplements/placebos will be offered to the enrolled children according to serial numbers provided by the producer.

**Clinical parameters.** All children will be visited weekly where we also will obtain morbidity data. At the visits, mothers will be asked about diarrheal illness (number and consistency of stools), symptoms of respiratory illness (cough, fast or difficult breathing), fever and physician visits. Weight and length/height will be measured at enrollment, every month and at the end of the 12 months period. Sick children that might require treatment will be referred to the study clinic for treatment.

**Laboratory parameters** Blood samples will be obtained at enrollment (baseline) and at the end of the 12 months follow-up period for each child. Three mL of blood will be collected into vials containing EDTA as anticoagulant. The plasma will be centrifuged at approx. 700 g at room temperature for 10 minutes, separated and transferred into storage vials, and stored at -70 degrees before analysis.

The blood samples will be analyzed for

1. Hemoglobin
   1. EDTA blood will be analyzed for hemoglobin by HemoCue B-Hemoglobin.
   2. For children where blood from the cubital vein is not obtained, capillary blood will be used for analysis of Hemoglobin
2. Plasma vitamin B12_,_ and plasma folate concentration will be estimated by microbiological assays using a chloramphenicol resistant strain of *Lactobacillus casei* and colistin sulphate resistant strain of *Lactobacillus leichmannii*, respectively ([23](#_ENREF_23), [24](#_ENREF_24)). We will also measure plasma concentrations of total Homocysteine and Methyl Malonic acid which are good markers vitamin B12 status.
3. The blood pellet will be analyzed for relative telomere length using quantitative real-time pcr.

**Measurements**

***Anthropometry.** Weights will be measured with a portable electronic scale that measures to the nearest 0.01 kg (Salter). Height and length will be measured according to standard guidelines as we have done previously. Both height and weight will be measured in their homes every month.

*Early Child development. All developmental tests will be carried out in a designated room at the field clinic (which is now used by the MAL-ED project for the same purpose). The main outcome of the neurodevelopment comparisons is the summary scores of the Bayley Scale of Infant and Toddler development 3^rd^. ed (Bayley-III) which is a comprehensive assessment tool of developmental functioning in infants and toddlers age 1-42 months. It is administered by a professional directly with the child. The tool provides information on functioning in different domains such as cognitive, communication and motor abilities. The Bayley-III represents the golden standard in assessment of this age-group and is widely used for research purposes.

The Ages and Stages Questionnaire – 3^rd^ ed. (ASQ-3) is an easily administered checklist of developmental status standardized for children 1-66 months. The screening system includes age-appropriate questionnaires for every two/three-month intervals; each questionnaire contains 30 items written in a simple language. The questionnaires are designed to be completed by caregivers, but can also be administered by a trained examiner in a “home procedure” such as in this project. The ASQ-3 has five subscales: Communication, Gross motor, Fine motor, Problem solving and Personal social. ASQ-3 is an easily administered and robust tool, which we will use at baseline and throughout the study as outlined below. The ASQ-3 scores will also be compared between the study groups.

The Ages and Stages Questionnaire: Social Emotional (ASQ:SE) is a screening tool on social and emotional behaviour in children 3-66 months ([25](#_ENREF_25)). It consists of eight age-appropriate questionnaires, and covers domains as self-regulation, compliance, communication, adaptive behaviours, autonomy, affect, and interactions with others. We will also compare the ASQ:SE scores between the study groups at end study.

Alarm Distress Baby Scale (ADBB) is an instrument developed to measure social behavior and withdrawal in infants 2-24 months old (http://www.adbb.net). The scoring is based on observation of the child, and the examination takes 10-15 minutes. Examiners will be trained and standardized to perform the ADBB observations according to existing standards of the ADBB program. The ADBB observations will be performed at the study clinic during the other assessments. We will compare the ADBB scores between the study groups at end study.

The Home Observation for the Measurement of the Environment (HOME) is a structured interview and observational measure of the psychosocial environment and quality of maternal responsiveness/parental practice (http://www.safeguardingchildren.co.uk/appendix-5.html). It has been used widely in international child development research and has shown to be predictive of intellectual abilities. The HOME will be performed in the homes of the children by trained field workers.

We have previously used the ASQ-3, ASQ:SE, HOME and the Bayley-3 in our projects in Nepal and India, and all these tests are currently used in our studies in Nepal. The ADBB will be new for the site, but in our opinion, this examination will be feasible within this site due to its extensive experience with infants and young children and their families. The ADBB training is standardized from the developers, and therefore suitable as one of the measurements in the study.

**Enrolment:** **Follow-up:**

6-11 mnd 12-17 mnd 18-23 mnd 4-4.5 years 7-7.5 years

ASQ-3 ASQ-3 ASQ-3 ASQ-3 WISC-IV

Bayley-3 Bayley -3 NEPSY-II

ASQ:SE ASQ:SE ASQ:SE

ADBB ADBB

Vagal tone

Eye tracking

HOME

**Heart rate variability (Vagal tone):** Vagal tone assessment is a non-invasive measure of vagally mediated heart rate variability which provides information regarding maturation of prefrontal cortical areas of CNS. Executive functioning in children such as action planning, behavior and emotion control are coordinate through prefrontal areas of brain. Prefrontal activation has been related to an individual’s ability to coordinate action with peripheral physiological (e.g., cardiovascular) activation and recovery, with executive functions, emotion regulation, and mental health. Prefrontal activation in children and individually different developmental trajectories can predict long-term health outcomes and cognitive functions in later years.

Methodology

The heart rate variability measures the difference between the average height of heart rate peaks and valleys caused by breathing (respiratory sinus arrhythmia). For this purpose, cardiac activity is measured by means of changes in blood volume in arteries and capillaries detected by photoplethysmographic (PPG) sensors in three finger caps attached to three fingers of one hand. The hardware used are caps put on the fingertips and a recording unit “Alive - Clinical Version©” (Somatic Vision Inc./CA, see picture). To determine cardiac activity in sufficient length for the analysis of vagal tone, the “Standards of measurement, physiological interpretation, and clinical use” published by the “Task Force of The European Society of Cardiology and The North American Society of Pacing and Electrophysiology” will be followed. These standards of measurement recommend a recording length of 5 minutes in which the participant sits conveniently in a chair and is instructed to relax and breathe normally for this period. To ensure relaxation and sufficient data quality, an additional time of 3 minutes is allowed. Offline data analysis follows international standards and published statistical procedures, using common software solutions.

Thus vagal tone assessment is correlated with the corresponding cognitive functions and low vagal tone has been identified as a risk factor for later psychopathology in adolescent and adulthood. The inclusion of vagal tone in the currently existing test protocols will add a physiological test variable complement to existing behavioral and report-based tests and will extend our possibilities to detect influences of malnutrition in developmentally very sensitive domains such as executive functions and emotional regulation. Early detection of vagal tone in children suffering from sub-optimal nutrition or other detrimental environmental influences can enable health care professionals to take action before pathologies related to dysfunctional prefrontal inhibition (e.g., mood disorders) become manifest. Moreover, there are limited data on effect of vitamin B12 supplementation on heart rate variability.

**Eye tracking:** The method of eye tracking involves the process of measuring points of gaze (where one is looking). For this purpose, a sensor bar with infrared diods will be placed below a video screen (e.g., a laptop or other monitor). On this screen, video clips can be presented showing logical tasks, how humans solve problems or interact, or other visual stimuli that have previously been used in studies on childhood brain development. All visual stimuli are harmless and without strong emotional content. The sensors measure gaze direction and fixation duration with high temporal resolution.

Methodology eye tracking.

No sensor or other device is attached to the participant/child, no movement restriction is required besides sitting on the parent’s lap, since head movements do not pose a problem for later analysis. Eye tracking in children and adolescents is safe and does not carry any health risk for the participant. It is based on infrared light emitting diodes that will pick up eye movements by assessing the visual reflection and change of light in the cornea. Participants sit usually 60 cm away from a computer screen on which they have to visually explore stimuli. The diodes are usually placed below the screen. Eye tracking has a long tradition and has been conducted in many clinical pediatric populations including studies investigating variations of normal brain development as well as neuropsychological assessment of developmental retardations or brain disorders. Side effects of eye-tracking are not reported as it is equivalent to simply watching a computer screen. A session can approximately 10 minutes. Eye-tracking sessions can be interrupted with numerous breaks.

A video clip from one of the most renowned laboratories in Sweden investigating neurocognitive development with eye-tracking method can be found in following link

<http://www.tobiipro.com/fields-of-use/infant-child-research/developmental-psychology/>

Hypotheses and Specific Aims

The temporal patterns of gaze durations recorded during eye tracking test will allow conclusions on the attentional processing and depth of cognitive processing and conceptual understanding. The results from eye tracking test will investigate variations of normal brain development as well as neuropsychological assessment of developmental retardations or brain disorders among Nepalese infants participating in vitamin B12 supplementation on growth and cognitive development.

**Training and quality control:**

Measuring neurodevelopment in all ages is challenging, particularly in low-income countries and in settings foreign to the one that the tests were developed for. We have therefore expanded the team to include two clinical psychologists (one Clinical neuropsychologist (Hysing) and one Clinical child and adolescent psychologist (Kvestad)) and one local pediatrician who specialize in child development (M Sthrestha). The salaries of Hysing and Kvestad will be covered by their Norwegian salaries.

Before study start the team will arrange a training workshop in Nepal for the psychologists responsible for assessing children in the study. The psychologists will be trained and standardized in performing the ASQ-3 “home procedure” and the Bayley – 3. The local paediatrician, dr. Shrestha will serve as the gold standard both during training and throughout the study. Ten % of all sessions will be scored by two examiners to ensure appropriate inter-observer agreement. The standardization and quality control procedures will be similar to the procedures in the previously mentioned, Thrasher funded study in New Delhi, India. In this study, the field supervisors achieved and maintained a high inter-rater agreement. In addition, we will videotape 5% of Bayley-3 and ASQ- 3 assessments, which will be checked by the supervising psychologists who will give prompt feedback to the assessors.

During the study period there will be regular Skype-meetings in the neurodevelopment team to discuss the progress in general and possible challenges faced. Hysing and Kvestad will visit the study site regularly, and additional training and standardizations will be organized. We will pay for their supervising visits by funds from this grant

**Assessment tools for the secondary outcomes**

**NEPSY-II** is a neuropsychological battery designed for the assessment of neurocognitive abilities in children aged 3-16 years ([26](#_ENREF_26)). We are currently applying this test in other studies in Bhaktapur, and are planning to use it in the follow-up phase of the project (i.e. after end of supplementation) as a secondary outcome of the trial. To avoid a floor effect (i.e. high proportion with very low scores), we will use the NEPSY-II when the children have reached 4 years of age together with the ASQ-3 and the ASQ:SE.

**WISC-IV** is an extensive test of general cognitive functioning for children 6 to 17 years old. It is individually administered, generates an IQ score and from 2012 available in an Indian version with Indian norms. To avoid a floor effect, this test will be administered when children have reach 7 years of age.

Intervention:

The intervention will be provided in sachets contain 20 grams of the supplements. Each sachet will provide 1 day of supplements. The composition of the lipid based supplement is shown in the table below.

|  |  |  |
| --- | --- | --- |

CG Rieber - Compact has produced the lipid-based supplement, which is very similar to the intervention that we have used before. This also contain several other vitamins at approximately 1 RDA. This is to ensure that the effect of vitamin B12 is not limited by deficiency of other nutrients. The composition of this multi-micronutrient mixture is based on the UNIMAP formulation (29).

Staff at CG Rieber Compact have labeled the supplements and the code that links the serial number of the packages to the group identity is being kept with them and with a scientist otherwise not involved in the trial. The researchers involved in this trial will not get this list until the analysis stage (see below).

**Dose of vitamin B12**

The RDA for vitamin B12 in children 1 – 3 years of age is 0.9 micrograms per day (Food and Nutrition Board, Institute of Medicine, National Academy of Sciences, 2002). We will give the child randomized to receive vitamin B12, 2 micrograms B12 per day.

This amount of vitamin B12 for 12 months does not constitute any health risk for the child. However, we will closely monitor possible adverse effects during follow-up. This will be done by observing the child for 20 minutes after the placebo or vitamin B12 was given. We will also ask specific questions regarding the children`s morbidity when visiting the homes.

Co-intervention.

All caretakers will be given dietary recommendations according to national guidelines.

Children with anemia (defined according to national guidelines) will be given peroral iron.

In short:

At enrollment and end study when we will measure Hemoglobin concentration (Hb) in all children.

Children with Hb 7 – 10 g/dL will be treated with peroral iron for 30 days and included if Hb > 10 g/dL.

Children with Hb < 7 g/dL will not be enrolled and referred to hospital

When enrolled (i.e. during the period when receiving placebo or vitamin B12):

In children with severe palmar pallor we will measure Hb.

Children with Hb 7 – 10 g/dL will be treated with peroral iron for 30 days (or longer if still anemic).

Children with Hb < 7 g/dL will be referred to hospital.

All children will receive 20 mg of zinc (dissolvable tablet, available locally) and oral rehydration solution if they fall ill with acute diarrhea.

**Treatment**

Children with pneumonia, dysentery or other illnesses will be treated according to the IMCI guidelines.

**Sample size calculations**

We have powered the study to have 90% power for most relevant outcomes, which are displayed in the table below. The standard deviations for BSID, ASQ and Z scores are derived from previous studies in Nepal and India and represent “a worst case scenario” in terms of variability of the outcome measurements. The power to detect a difference of 4 BSID points is almost 80% even with a SD of 17. The expected SD of the BSID is normally 15.

| **Power calculations –assuming a group size of 300** | | | | |  |
| --- | --- | --- | --- | --- | --- |
|  |  | **Effect** | **SD** | **Power** |  |
|  | **BSID** | 4 | 15 | 87.3 |  |
|  |  | 5 | 15 | 97.2 |  |
|  |  | 4 | 17 | 78.1 |  |
|  |  | 5 | 17 | 92.8 |  |
|  | **ASQ** | 15 | 60 | 82.8 |  |
|  |  | 20 | 60 | 97.2 |  |
|  | **Z scores** | 0.2 | 0.75 | 87.3 |  |
|  |  | 0.25 | 0.75 | 97.2 |  |
|  | **Hemoglobin** | 0.5 | 1.5 | 97.2 |  |
|  |  | 0.4 | 1.5 | 87.3 |  |
|  |  | 0.3 | 1.5 | 64.2 |  |
|  | BSID: Bayley Scales of Infant Development, version III | | | | |
|  | ASQ: Ages and Stages Questionnaire, version III | | | | |
|  | Z scores: change in Z scores from baseline to end study | | | | |
|  | Hemoglobin: Hemoglobin concentration in grams per liter | | | | |

An effect size of 4 points corresponds to approx. .25 to .3 SD, and is a commonly used figure in such trials. We would not expect that a single intervention has an effect beyond this magnitude. The most important time of brain development is before the child reaches her 2^nd^ birthday and small differences in brain functioning at this stage can result in larger and more profound differences later in life.

**Supervision and ensuring compliance of the daily follow-up**

Supervisors will organize the work-flow from the central office. They will also monitor the fieldworkers performance in the field by undertaking supervised and non-supervised visits to the field. In a random sample of the scheduled follow ups the supervisors will follow the field workers and observe their performance and interaction with the study participants. In the “unsupervised visits” they will visit the study participants after the fieldworkers have completed their tasks and ask questions about their performance, behavior and when possible, attempt to collect the same information as the field workers. We will undertake such supervised and unsupervised visits in 2% of all scheduled visits.

**Data management and analysis**

All forms will be checked manually by supervisors for completeness and consistency. The data will then be double entered at the field clinic into appropriate databases with computerized range and consistency checks. In this process there will be continuous data cleaning. Comparison of baseline features including vitamin B12 status will be made to confirm comparability across treatment groups.

We will use the statistical software packages Stata and SAS to analyze the data. A detailed plan for analysis of the main outcomes will be made before the list that links the child identity numbers to the treatment groups are obtained by the researchers. Data cleaning, definition of outcome variables, exclusion of cases as well as programming of scripts in the statistical packages will also be done before the analysis-files are merged with the randomization lists. The intervention groups will be coded in a way so that the researchers do not know the group identities until the main analyses are finished. In these analyses there will be one variable denoting the group identity. The analyses will be planned and undertaken in a joint workshop attended by the involved scientists. All analyses will initially be done on an intent-to-treat- basis, to adjust for potential baseline differences we will use multiple regression models.

**Dissemination of the results**

The results of this trial will be published in international biomedical journal with peer review. Nepali scientists will be responsible for the reports describing the main outcomes of the trial. The scientific staff in Nepal is also responsible for rapid dissemination to local and national health authorities. This process will start as soon as possible after the meeting where the analysis workshop is held.

## Risk of the project

A team of Nepali and Norwegian researchers that have been working together for more than 15 years will undertake this project. The main competence of this team is randomized controlled field trials in young South Asian children. Growth and cognitive development have been studied in many of our projects (see individual CVs). Our research site in Bhaktapur, Nepal was established in 1997 and was in 2007 selected as one of 8 international sites for the MAL-ED study [The Etiology, Risk Factors and Interactions of Enteric Infections and Malnutrition and the Consequences for Child Health and Development Project (MAL-ED) is carried out as a collaborative project supported by the Bill & Melinda Gates Foundation]. Two of summary scores of the MAL-ED study are growth and cognitive development. Thus, we have experience in using the main tools for this project. The team of supervisors, field workers and key scientific staff has been working together for more than a decade, which substantially reduces the risk for ”human friction”.

We will take the following measures to ensure that our measurements are valid.

1. The staff will go through extensive training before study start. Only those who reach a certain level of skills in measuring growth and who are interacting with the staff properly will be allowed to work as field workers. They will be assessed prior to study start and intra and inter observer (comparison with a gold standard) variability will be measured to quantify their skills. Such evaluation of the staff will also take place on regular intervals throughout the trial.
2. A manual with the definitions and protocol will be kept in the field office and the field workers will at all times carry a short version of the manual describing their tasks.
3. Supervised (observation of the field workers at work) and non-supervised (assessment of the quality of the performance of the field worker after the field worker have made a home visit) will be undertaken in 5% of all household visits. These quality checks will be done by the supervisors and selected randomly among the enrolled children. On the non-supervised visits, the supervisors will ask questions on the behavior of the field workers. This system ensures that misclassification of the exposure is kept to a minimum by ensuring that dispensing is being done.

We are working closely with local and national health authorities to ensure good compliance with the community and the public health authorities. In the field site we have been running a clinic for 15 years. We have ensured that there have been at least 2 medical doctors at any time to provide free treatment and immunizations to children in the community. We believe that this long-standing relation ensures excellent compliance with the community and increases the likelihood of success of the project.

Ethical considerations

Clearance from the medical ethics committee at the Institute of Medicine and the regional ethical committee of Norway that is responsible for the supervising institution in Norway will be obtained before the study is initiated. All aspects of the study will be in agreement with the latest version of the Helsinki declaration and we will undertake and report the study in accordance to the guidelines of the “Consort” statement ([27](#_ENREF_27)). The study will also be registered with clinicaltrials.org.

Mitigation

During the project period, we will continue to staff a field clinic where all eligible children will be offered free examination and treatment for common conditions including diarrhea and ALRI. Children will be treated for common infections according to IMCI guidelines.

***Implementation strategy:***

**Inclusion criteria**

- Age: 6 to 11 months
- Either sex
- Likely to reside in area for next 12 months
- < -1 z scores length for age

**Exclusion criteria**

- Severe systemic illness requiring hospitalization

- Severe malnutrition, i.e. weight for height < -3 z of the WHO standard for this age group. For ethical reasons these children require micronutrient supplementation and adequate medical care.

- Lack of consent

- Taking B vitamin supplements that include vitamin B12.

- Severe anemia (Hb < 7 g/dL). This would be a temporary exclusion and the children will be enrolled if this is successfully treated.

- Ongoing acute infection with fever or infection that requires medical treatment. This would be a temporary exclusion and the children will be enrolled after recovery.

**Figure 3: Study timeline**

**Study Procedures**

| **Activity** | **When** | **By Whom** |
| --- | --- | --- |
| Anthropometry (weight, length) | At enrollment, every month, 12 months post enrollment | Field workers |
| Blood sample collection | At enrollment, 12 months post enrollment | Physicians, Phlebotomist |
| Vitamin B12, Placebo supplementation | Daily supplementation (12 months) | Field worker; dispensing |
| Morbidity ascertainment | Weekly throughout study period (12 months) | Field worker; morbidity ascertainment |
| Referral to clinic | Whenever predetermined criteria are present on a morbidity visit | Field workers |
| Referral to physician for screening for clinical pneumonia | All cases having cough and high respiratory rates (≥6 months to <12 months RR ≥45, ≥12 months RR ≥35) or lower chest indrawings | Field workers |

| **Activity** | **When** | **By Whom** |
| --- | --- | --- |
| Diagnosis of clinical pneumonia | Through all cases referred by Field worker and spontaneous visits to clinic | Physician |
| Diagnosis of other illnesses | Throughout study period | Physician |
| Spontaneous visits to clinic by mothers | Whenever they feel child needs to see a doctor | Physician |

**Outcome measures**

| **Activity** | **When** | **By Whom** |
| --- | --- | --- |
| Anthropometric status | Weight for age, weight for height, height for age at enrollment and after 12 months of supplementation | Field workers |
| Biochemical assays | Hemoglobin, plasma folate, B12, tHcy and MMA at enrollment and after 12 months of supplementation | Hemoglobin will be measured on site. Plasma will be stored at -70 and analyzed in Norway (www.bevital.no) |
| Neurodevelopment | Bayley Scales of Infant and Toddler development 3^rd^. ed. at baseline and after 12 months of supplementation  Ages and Stages questionnaire 3^rd^ ed. at baseline and after 6 and 12 months of supplementation  Ages and Stages questionnaire: Socio- Emotional at baseline and 12 months of supplementation  Alarm Distress Baby Scale: At baseline and after 12 months of supplementations  HOME: at baseline | By trained psychologist |

**Survey**

The study will be conducted in Bhaktapur municipality and the adjoining districts. We have been conducting clinical trials and observation studies in children and fertile women in this area since 1997. Currently we undertake observational studies that all involve cognitive testing of young children. These studies will end in 2013 and early 2014. Prior to study start we will do a household survery and identify all households with pregnant women and infants.

## Screening and Consent Procedures

Eligible and willing children will be brought to the clinic along with their caregivers. Here they will be screened by the physician/supervisor for eligibility criteria. Only one child will be enrolled from each household.

Prior to enrollment, informed consent will be obtained by the physician/supervisor. An information sheet will be read out to the caregiver. The caregiver will sign the consent form; if the caregiver is non-literate, the consent will be witnessed by an impartial witness. A list of witnesses will be kept.

Prior to initiating supplementation, socioeconomic details of the enrolled child will be collected, the HOME inventory questionnaire will be completed, weight and length will be taken, and the ADBB form filled in. The baseline blood sample will be collected. The first dose vitamin B12 or placebo will be administered to the child. The supplement will then be handed over to the mother.

Field workers in charge of supplement administration will visit the child’s home daily on all working days and administer the supplement to the child. On Saturday (which is a public holiday in Nepal), instructions will be left with the mother to dispense the same to the child. The supplements will be replaced by fresh jars at home by the field worker periodically.

**Morbidity visits**

A separate team of trained fieldworkers will visit the households once every week. During these visits they will ask for day-vice symptoms of illnesses, morbidity, visits to other treatment facilities, hospitalizations for the past 7 days. The fieldworkers will also undertake a physical examination of the children that includes assessment of dehydration (if the child has diarrhea), respiratory rate count (if the child has symptoms of lower respiratory illness), and measure temperature (if caretaker reports fever).

**Visits of enrolled children to the study clinic**

These will occur under the following circumstances.

- whenever the mother brings the child to the clinic because she feels the child needs to see a doctor.
- the child is referred to the clinic by a field worker at a morbidity visit.
- physician requests follow up for an illness. Standard treatment for morbidity based on national guidelines will be prescribed.

Verbal autopsies will be conducted by the physicians for all deaths that occur in enrolled children as early as possible after the child’s death.

If an enrolled child is hospitalized, the field worker will record the hospitalization details.

**End study activities**

At end study (12 months), in all children, early child development tests by the psychologist(s), anthropometric measurements by field worker, and a blood sample by a physician (or laboratory technician).

| **Human Subjects** |
| --- |

Six hundred children will be enrolled in this study. There are no indications from the literature that the intervention is unsafe for the children. We will provide 2 µg of vitamin B12. This is the same dose as we provided in the previously mentioned study in 1,000 North Indian children. In this trial, where we monitored immediate adverse events and morbidity closely, vitamin B12 administration was not associated with any discomfort or harm to the children. We will follow the children closely for possible side effects of the intervention in this trial also. For the first week of administration, field workers will wait for 30 minutes after the supplement has been given to observe any potential side effects.

The lipid-based vehicle that we will deliver the vitamin and co-interventions in has also been used in several populations with no associated health risk. The vehicle contains, however, traces of peanuts and may pose some hazard to children who are allergic to peanuts. We will therefore ask the caregivers if the children have had an allergic reaction to peanuts and exclude these children from the study. It should be noted that in the study in North Indian children there were no reports of peanut allergy and no child had an allergic reaction because of the supplements that we gave.

All children that are enrolled in this study will be offered free treatment in the field clinic and the field staff will follow them closely. Thus, participation in this trial will benefit the children.

The research will follow International, Nepali and Norwegian ethical guidelines and the study will not be started before we have approval from the relevant ethical review boards in these countries. The trial will also be registered in clinicaltrials.gov.

Participation will also be voluntary and if the caregivers chose to withdraw their child, they are free to do so at any time without loosing their benefits that we have offered them (except he close follow up)

We will follow international standards of keeping the computerized data. Only the involved researchers will have access to the data and the personal information that includes names, addresses or GPS coordinates will not be kept with the files used for statistical analyses. These lists will be stored electronically on devices not connected to the internet.

| **Literature References** |
| --- |

1. Black MM. Micronutrient deficiencies and cognitive functioning. J Nutr 2003;133(11 Suppl 2):3927S-31S.

2. Allen LH. Folate and vitamin B12 status in the Americas. Nutrition reviews 2004;62(6 Pt 2):S29-33; discussion S4.

3. Taneja S, Bhandari N, Strand TA, et al. Cobalamin and folate status in infants and young children in a low-to-middle income community in India. Am J Clin Nutr 2007;86(5):1302-9. doi: 86/5/1302 [pii].

4. Shane B, Stokstad EL. Vitamin B12-folate interrelationships. Annual review of nutrition 1985;5:115-41.

5. Selhub J, Morris MS, Jacques PF, Rosenberg IH. Folate-vitamin B-12 interaction in relation to cognitive impairment, anemia, and biochemical indicators of vitamin B-12 deficiency. Am J Clin Nutr 2009;89(2):702S-6S. doi: ajcn.2008.26947C [pii]10.3945/ajcn.2008.26947C.

6. Smith AD, Refsum H. Vitamin B-12 and cognition in the elderly. Am J Clin Nutr 2009;89(2):707S-11S. doi: 10.3945/ajcn.2008.26947D.

7. Bjorke Monsen AL, Ueland PM. Homocysteine and methylmalonic acid in diagnosis and risk assessment from infancy to adolescence. Am J Clin Nutr 2003;78(1):7-21.

8. Schneede J, Dagnelie PC, van Staveren WA, Vollset SE, Refsum H, Ueland PM. Methylmalonic acid and homocysteine in plasma as indicators of functional cobalamin deficiency in infants on macrobiotic diets. Pediatr Res 1994;36(2):194-201.

9. Louwman MW, van Dusseldorp M, van de Vijver FJ, et al. Signs of impaired cognitive function in adolescents with marginal cobalamin status. Am J Clin Nutr 2000;72(3):762-9.

10. Siegel EH, Stoltzfus RJ, Kariger PK, et al. Growth indices, anemia, and diet independently predict motor milestone acquisition of infants in south central Nepal. J Nutr 2005;135(12):2840-4. doi: 135/12/2840 [pii].

11. Strand TA, Taneja S, Ueland PM, et al. Cobalamin and folate status predicts mental development scores in North Indian children 12–18 mo of age. 2013. doi: 10.3945/ajcn.111.032268.

12. Kvestad I, Taneja S, Kumar T, Mohan M, Bhandari N, Strand TA. Administration of Vitamin B12 and Folic acid improves developmental scores in young North Indian children. Manuscript 2013.

13. Thompson RA, Nelson CA. Developmental science and the media. Early brain development. The American psychologist 2001;56(1):5-15.

14. Walder D. J., Sherman J. C., Pulsifer M. B. Neurodevelopmental Assessment. . Evidence-Based Practice in Infant and Early Childhood Psychology 2009:167-205.

15. Anderson V, Spencer-Smith M, Leventer R, al e. Childhood brain insult: can age at insult help us predict outcome? Brain 2009;132:45-56.

16. Cusick SE, Georgieff MK. Nutrient supplementation and neurodevelopment: timing is the key. Arch Pediatr Adolesc Med 2012;166(5):481-2. doi: 10.1001/archpediatrics.2012.199.

17. Dagnelie PC, van Staveren WA. Macrobiotic nutrition and child health: results of a population-based, mixed-longitudinal cohort study in The Netherlands. Am J Clin Nutr 1994;59(5 Suppl):1187S-96S.

18. Dror DK, Allen LH. Effect of vitamin B12 deficiency on neurodevelopment in infants: current knowledge and possible mechanisms. Nutrition reviews 2008;66(5):250-5. doi: 10.1111/j.1753-4887.2008.00031.x.

19. Calvaresi E, Bryan J. B vitamins, cognition, and aging: a review. The journals of gerontology Series B, Psychological sciences and social sciences 2001;56(6):P327-39.

20. Eilander A, Gera T, Sachdev HS, et al. Multiple micronutrient supplementation for improving cognitive performance in children: systematic review of randomized controlled trials. Am J Clin Nutr 2010;91(1):115-30. doi: 10.3945/ajcn.2009.28376.

21. Faber M, Kvalsvig JD, Lombard CJ, Benade AJ. Effect of a fortified maize-meal porridge on anemia, micronutrient status, and motor development of infants. Am J Clin Nutr 2005;82(5):1032-9.

22. Bjørke Monsen AL, Ueland PM. Homocysteine and methylmalonic acid in diagnosis and risk assessment from infancy to adolescence. Am J Clin Nutr 2003;78(1):7-21.

23. O'Broin S, Kelleher B. Microbiological assay on microtitre plates of folate in serum and red cells. J Clin Pathol 1992;45(4):344-7.

24. Kelleher BP, Walshe KG, Scott JM, O'Broin SD. Microbiological assay for vitamin B12 with use of a colistin-sulfate-resistant organism. Clin Chem 1987;33(1):52-4.

25. Squire M, Bricker D, Twombly E. Ages and stages questionnaires: Social-emotional. Baltimore: Brookes publishing, 2002.

26. Brooks BL, Sherman EM, Strauss E. Test Review: NEPSY-II: A DevelopmentalNeuropsychological Assessment, Second Edition. Clinical Neuropsychology 2010;16:80-1001.

27. Moher D, Schulz KF, Altman DG. The CONSORT statement: revised recommendations for improving the quality of reports of parallel-group randomised trials. Lancet 2001;357(9263):1191-4.

28 Torsvik, I., Ueland, P. M., Markestad, T., & Bjorke Monsen, A.-L. (2013). Cobalamin supplementation improves motor development and regurgitations in infants: results from a randomized intervention study. *American Journal of Clinical Nutrition*. doi:10.3945/ajcn.113.061549

29 Christian, P. (2010). Micronutrients, birth weight, and survival. *Annual Review of Nutrition*, *30*, 83–104. doi:10.1146/annurev.nutr.012809.104813

| **Collaborative Arrangements** |
| --- |

The group of researchers behind this project is from the Centre for International Health and Faculty of Psychology at the University of Bergen, and from the Institute of Medicine at Tribhuvan University. We have established a project management team (PMT), which we have divided into three domains. A scientist from the “neurodevelopmental assessment” domain will be the first author on the report (publication) on neurodevelopment (primary objective 1) and scientist from the “Field implementation” domain will be the first author on the reports on the effect of vitamin B12 supplementation on growth and hemoglobin concentration. As in our previous projects, we will attempt to secure funding for junior, key Nepali scientists so they can pursue master or PhD degrees at the University of Bergen using data collected in this project.

**Project management team (PMT):**

Current members:

**Coordinators:** IoM: Prakash S Shrestha (MD - pediatrics), UoB: Tor A Strand (MD/PhD - Nutrition)

**Field implementation:** Ram K Chandyo (MD/PhD pediatrics), Manjeswhori Ulak (MD pediatrics)

**Cognitive assessment:** IoM: Merina Shrestha (MD Pediatrics), UoB: Ingrid Kvestad (Phd - Clinical child and adolescent psychologist), Mari Hysing (Phd – Clinical Neuropsychologist).

The project management team will be expanded to include scientists with laboratory experience.

The principal investigator will visit the field site 3-4 times every year during the data collection period. Most of the travels for this purpose will be covered from other sources. In addition, we will have regular Skype meetings. Initially these will be quite frequent, i.e. every week, after the study has started we will schedule monthly meetings. The calls will include at least one member from of each of the domains of the PMT.

The questionnaires, computer entry system, and standard operating procedures will be developed jointly by the PMT.

The members of the neurodevelopmental assessment team are currently working together on other studies on the assessment of neurodevelopment and cognition in early childhood.
