# Supplementary material for: Effects of vitamin B12 supplementation on neurodevelopment and growth in Nepalese Infants: A randomized controlled trial
Source: PLoS Med. 2020 Dec 1;17(12):e1003430. doi: 10.1371/journal.pmed.1003430 (PMC7707571; doi:10.1371/journal.pmed.1003430)
Supplement: S3 Text — March 2018. (DOCX) [file pmed.1003430.s008.docx]

**Plan of Analysis**

**The effect of vitamin B12 supplementation in Nepalese infants on growth and development:**

**study protocol for a randomized controlled trial**

**version 1, March 2018**

The analyses will be planned and undertaken in a joint workshop attended by the involved scientists.

All analyses will be done on an intention-to-treat-basis. All randomized participants will be included in the analyses if the relevant outcome variables have been collected.

The main outcomes are continuous and expected to be normally distributed. We will check all continuous dependent variables for normality by inspecting histograms.

We will use the change in Bayley-III scores from baseline to the end of the study in separate analyses where B12 supplementation is the main exposure. We will compare the mean Bayley-III scores (total score and scores on the subscales: cognitive, language, and motor, with the motor scale analyzed both separately for fine and gross motor development and as a composite measure) between the vitamin B12 group and the placebo group.

Change in raw scores from baseline to end study (delta scores) will be the main comparison between the vitamin B12 group and the placebo group in the statistical analyses

A fully specified statistical analysis plan is provided below

**Main outcome 1:** Neurodevelopment: Bayley

- - - - Delta raw score
        - **Cognitive**
        - **Language**

Expressive and Receptive

- - - - - **Motor**

Fine and Gross

- - - - End study Scaled Scores will also be shown
        - **Cognitive**
        - **Language**

Expressive and Receptive

- - - - - **Motor**

Fine and Gross

**Main outcome 2**: Growth: length, weight, HAZ, WAZ,

- Delta cm from baseline to end study
- Delta kg from baseline to end study
- Delta HAZ from baseline to end study
- Delta WAZ from baseline to end study
- End study length (cm), (kg), HAZ, WHZ, WAZ

**Main outcome 3:** Hemoglobin (Hb) concentration

- Delta Hb
- End study Hb
- End study anemia prevalence

Descriptive: compliance, adverse events and perception

**TABLES:**

Table 1: Baseline features – by Placebo and B12 groups

Table 2: Compliance (form P and J) – No of days taken, divided doses, how given, how much given. Biochemical response to the supplementation. Baseline and end study total homocysteine, methyl malonic, and cobalamin concentration. Combined indication of vitamin B12 status.

Table 3: Adverse events (vomiting etc.) and caregiver’s perception of the supplement use.

Table 4: Main outcome neurodevelopment – Delta raw scores – and scaled end study scores

Table 5: Main outcome growth – Delta cm and kg, end study cm, kg, HAZ, WHZ, WAZ, Head circumference. Main outcome Hemoglobin - Delta HB, End study HB, End study Anemic

**FIGURES:**

Figure 1: Flow chart

Figure 2: Forest plot – neurodevelopment (delta cognitive, language, motor) by subgroup

Figure 3: Forest plot – growth (delta cm and Hb) by subgroup

Predefined subgroups: Stunting, Wasting, Anemia, low birth weight (cut off at 2500g), B12 status (cb12<-.5), excl.breastfeeding at 3 months.

**Statistical analyses**

All the listed outcomes are expected to be normally distributed and will be compared using students t-test or linear regression. IWe will present differences in proportions for dichotomous outcomes. For these analyses, we will use generalized linear models with log links. For the subgroup analyses we will use linear regression models adjusting for socioeconomic status, maternal education, age of mother, and other relevant variables expected to be associated with growth and neurodevelopment. If relevant baseline differences are detected from table 1, we will also adjust the effect estimates for these variables in the regression models and present the adjusted effect in the main text of the manuscript**.**
