# Supplementary material for: Effects of vitamin B12 supplementation on neurodevelopment and growth in Nepalese Infants: A randomized controlled trial
Source: PLoS Med. 2020 Dec 1;17(12):e1003430. doi: 10.1371/journal.pmed.1003430 (PMC7707571; doi:10.1371/journal.pmed.1003430)
Supplement: S4 Text — October 2019. (DOCX) [file pmed.1003430.s009.docx]

**Plan of Analysis**

**The effect of vitamin B12 supplementation in Nepalese infants on growth and development:**

**study ‘ for a randomized controlled trial**

**version 2.1, October 2019**

The analyses will be planned and undertaken in a joint workshop attended by the involved scientists.

All analyses will be done on an intention-to-treat-basis. All randomized participants will be included in the analyses if the relevant outcome variables have been collected.

The main outcomes are continuous and expected to be normally distributed. We will check all continuous dependent variables for normality by inspecting histograms.

We will use the change in Bayley-III scores from baseline to the end of the study in separate analyses where B12 supplementation is the main exposure. We will compare the mean Bayley-III scores (scores on the subscales: cognitive, language, and motor) between the vitamin B12 group and the placebo group.

Change in raw scores from baseline to end study (delta scores) will be the main comparison between the vitamin B12 group and the placebo group in the statistical analyses.

Composite variables:

Weight for age (WAZ), weigh for length (WLZ), and length for age (LAZ) z-scores will be calculated using the 2006 growth curves from the World Health Organization.

A composite variable for the vitamin B12 status will be calculated using the method suggested by Fedosov and coworkers (based on the plasma concentrations of cobalamin, methyl malonic acid, total homocysteine, and folate).

A fully specified statistical analysis plan is provided below

**Main outcome 1:** Neurodevelopment: Bayley

- - - - Delta raw score
        - **Cognitive**
        - **Language**

Expressive and Receptive

- - - - - **Motor**

Fine and Gross

- - - - End study Scaled Scores will also be compared between the study groups
        - **Cognitive**
        - **Language**

Expressive and Receptive

- - - - - **Motor**

Fine and Gross

**Main outcome 2**: Growth: length, weight, LAZ, WAZ,

- Delta cm from baseline to end study
- Delta kg from baseline to end study
- End study length (cm), (kg), LAZ, WLZ, WAZ

**Main outcome 3:** Hemoglobin (Hb) concentration

- Delta Hb
- End study Hb

**TABLES:**

Table 1: Baseline features – by Placebo and B12 groups

Table 2: Biochemical response to the supplementation. Baseline and end study total homocysteine, methylmalonic acid, and cobalamin concentrations. Combined indication of vitamin B12 status. Biomarkers will be transformed if not normally distributed and the back-transformed effect estimates will be presented by study group (if possible). The relationship between changes in vitamin B12 status will be shown according to vitamin B12 status (3cB12) at baseline. This will be done by performing a kernel-weighted local polynomial regression of delta 3cB12 on baseline 3cB12 values by treatment group and depict these dose-response graphs of the predicted values. The concentrations of cobalamin, tHcy, and MMA will also be compared between the study groups in all infants and according to 3cB12 categories (“possible deficient”, “low” and “adequate”) at baseline.

Table 3: Main outcome neurodevelopment – Delta raw scores – and scaled end study scores

Table 4: Main outcome growth – Delta cm and kg, end study cm, kg, LAZ, WLZ, WAZ,. Main outcome Hemoglobin - Delta HB, End study HB

**FIGURES:**

Figure 1: Flow chart

Figure 2: The association between vitamin B12 status at baseline and change in vitamin B12 status from baseline to end study by study group.

Figure 3: Forest plot – neurodevelopment (delta cognitive, socioemotional, fine motor, gross motor, expressive language and receptive language) by subgroup

Figure 4: Forest plot – growth (delta LAZ and WAZ) by subgroup

Predefined subgroups: Stunting, Wasting, Underweight, Anemia, low birth weight (cut off at 2500g), B12 status (cb12<-.5), excl. breastfeeding at 3 months.

**Statistical analyses**

All the listed primary outcomes are expected to be normally distributed and will be compared using students t-test or linear regression. We will present differences in proportions for dichotomous outcomes. For these analyses, we will use generalized linear models with log links. For the subgroup analyses we will use linear regression models adjusting for socioeconomic status, maternal education, age of mother, and other relevant variables expected to be associated with growth and neurodevelopment. If relevant baseline differences are detected from table 1, we will also adjust the effect estimates for these variables in the regression models and present the adjusted effect in the main text of the manuscript**.**
