# Supplementary material for: Effects of vitamin B12 supplementation on neurodevelopment and growth in Nepalese Infants: A randomized controlled trial
Source: PLoS Med. 2020 Dec 1;17(12):e1003430. doi: 10.1371/journal.pmed.1003430 (PMC7707571; doi:10.1371/journal.pmed.1003430)
Supplement: Attachment — Submitted filename: ResponsesPloSMedicine_R1.pdf [file pmed.1003430.s011.pdf]

Dear Editor,

Thank you for the positive reviews and constructive feedback to our manuscript. The manuscript has been revised taking all of the comments into account. Below is a point-by-point response (in *italics*) to each of the comments.

Bergen, Norway
September 16, 2020
Tor A. Strand
On behalf of the authors.

Requests from the editors:

- If possible, please present the title per the PLOS Medicine style, this should present the study objective/question in the first part of the title and then the study design (eg, "A randomized controlled trial," "A retrospective study," "A modelling study," etc.) in the subtitle (ie, after a colon).

*Response: The title has been changed and now reads: "**The effect of vitamin B_{12} supplementation on neurodevelopment and growth in Nepalese infants: A randomized controlled trial**"*

- In the last sentence of the Abstract Methods and Findings section, please include a brief note about any key limitation(s) of the study's methodology.

Response: The Abstract has been altered (line 22).

- Please complete the CONSORT checklist (http://www.consort-statement.org/), designed to aid reporting of randomized trials, and ensure that all components of CONSORT are present in the manuscript. We'd suggest noting in the methods section that the trial is reported in line with CONSORT; please upload the completed CONSORT checklist as a supplementary file with your resubmission.

Response: An updated consort checklist has been uploaded

- Per the CONSORT guidance, we'd recommend that the abstract and Results section should present the estimates of effect size (with 95% CI's) on the main outcomes for the trial so that readers can get a sense of what the range of effects are that are plausible with the data - rather than just the narrative summary that there is no effect.

Response: We have included the key effect sizes and corresponding 95% Cis in the Abstract (lines 19-22) and results.

The following text has been added to the results section (lines 259-263: "For example, the composite cognitive and language scores were 0.73 (95% CI: -0.55 to 2.02) points lower in the vitamin B_{12} group compared to the placebo group. There was also no effect on growth or hemoglobin

concentration (Table 3). Children in both groups grew on average 12.5 cm (SD: 1.8) and the mean difference was 0.20 (95% CI: -0.23 to 0.63) cm. The mean difference in hemoglobin concentration between the groups was 0.02 (95% CI: -1.33 to 1.37) g/dL."

- It would be helpful if the authors could say something about possible harms (adverse effects) with the supplementation in the trial - the trial registration suggests that adverse effects would be monitored but the results section does not seem to present any data for this. Obviously in trials both benefits and risks are important.

Response: We agree. Please see lines 266-267 and the adverse effect table that is included as a supplementary table (supplementary table 3).

- It would also be good to include some discussion about possible external validity (generalisability) of the trial findings given that, as noted by reviewers, the children in the study are marginally stunted.

Response: We have included a section on external validity in the Discussion (line 329 onwards). "Restricting the participants to mildly stunted children increased the internal validity and statistical power of our study at the expense of the external validity. In other words, our recruitment strategy reduced the generalizability of our results, which is a limitation. It should be noted that enrolling all infants from the community would result in a larger proportion with adequate vitamin B_{12} status and, consequently, more children who would not respond to the intervention. A design ensuring higher external validity could accordingly attenuate a potential effect and, at the same time, increase the variability, both contributing to reduced statistical power. Reduced statistical power increases the risk of false-negative results (type II errors), which particularly question null findings such as here. In addition, our study was designed to measure the effects of vitamin B_{12} supplementation on several outcomes across several subgroups, which ensured we did not overlook relevant short-term clinical outcomes.

An important assumption of our study was that the inclusion criteria ensured we targeted those who would benefit from supplementation. It is possible, however, that these mildly stunted children would suffer from deficiencies of other growth-limiting nutrients. We believe this potential bias was accounted for by providing other vitamins and minerals, by treating common infections, and by giving zinc for diarrhea. "

Comments from the reviewers:

Reviewer #1: This is a statistical review of manuscript PMEDICINE-D-20-00074_R1. The manuscript is well written and easy to follow. Some clarifications are needed in the statistical section.

Major comments:

- Statistical analyses section: "Differences in proportions were calculated using generalized linear models (GLM) with the binomial distribution family and identity

link function". Please could you clarify with you did not use one of the most common link functions for binary data (i.e. logit, cloglog, etc). The identity link is rarely used.

Response: Thank you for seeing this. We used the binomial distribution family and the identity link function to calculate differences in proportions. However, the results of the analyses were removed from the manuscript text just before submission, we changed the description of the statistical methods to not include the description of binomial distribution family and identity link function.

- Statistical analyses section: "For these analyses we used crude and multiple GLMs with the gaussian distribution family and identity link function adjusting for a set of predefined potential confounders". Are you referring to continuous endpoints or binary endpoints? It is not clear currently, as this sentence follows the previous sentence that cover difference in proportions.

Response: Please see the response above, the statistical analysis text has now been changed (line 216 onwards).

Minor comment:

- Abstract: please specify what was percentage of loss to follow up (26/600=4.3%). Also, 2 children could not be included in the analysis. I think it might be worth stating the size of your complete data set and stating how it compares to the original 600 children as percentage.

Response: The changes have been made to the abstract. We did not mention the 2 children who could not be included in the neurodevelopmental assessment because we had data for the other outcomes.

Reviewer #2: This is a clearly written paper describing a blinded RCT of Vitamin B12 supplementation in Nepalese infants. The primary outcomes were neurodevelopment, growth and haemoglobin concentration. The authors have succinctly presented the rationale for the study in the introduction. The methods are clearly described but would benefit from the inclusion of the CONSORT checklist,

Response: Thank you, the checklist has been included.

- In the supplementary section. It would be useful to include information about trial registration.

Response: Included (lines 28-29)

- The authors are to be commended in the presentation of the results which are presented primarily as mean differences with 95%CI, eliminating the need to include p-values.

Response: Thank you.

- The intervention had no effect on the outcomes, even after sub-group analysis was undertaken. The authors complete the paper with a concise but well thought-out discussion. The inclusion of supplemental tables and figures are useful for those who are interested in more detail. A minor point is the use of tense; sometimes present and sometimes past.

Response: Thank you for the advice, the text has now been amended.

- In conclusion, this paper provides an exemplary model for how clinical researchers can present data from a RCT.

Response: Thank you.

Reviewer #3: The manuscript under review by Strand et al. reports on a randomized controlled trial of B12 supplementation to Nepalese infants and potential effects on metabolic and developmental outcomes.

- The manuscript is nicely written and generally fits the scope of PLoS Medicine, however, the results as presented may not be sufficient to be considered a substantial advance over existing knowledge. While they are clear and support the conclusion of the authors, the results may not be applicable to the general population as the study focus concentrated on marginally stunned children with a specific treatment dose of B12.

Response: Thank you, for raising this important point. The generalizability, or external validity of our study, was affected by the strict inclusion criteria. We deliberately prioritized internal validity over external validity. By using strict inclusion criteria and thus targeting infants who are most likely to benefit from supplementation, we limited the possibility of potential B_{12} effects being diluted by infants who were not expected to benefit from supplementation. Furthermore, the inclusion criteria also ensured that the variability between the study participants was minimized, which contributed to increased statistical power. We believe that reduced generalizability is particularly relevant if we find an effect, on the contrary, when no effect was observed, it is important to know that there was sufficient statistical power (to reduce the risk of false negative results, i.e. type II errors). We are glad the reviewer raised this issue and have added a section concerning limited generalizability towards the end of the Discussion (line 329 onwards).

- While there is no question about the validity of this approach, it may have not resulted in improvements for neurodevelopment and growth but the dose was sufficient to improve selective B12 biomarkers in the deficient infant subgroup indicating that there could be a benefit of B12 supplementation.

Response: Thank you. The response in biomarker concentrations demonstrated that there was a beneficial metabolic effect of supplementation, which may not be captured by the relatively crude clinical endpoints. As the reviewer points out, this metabolic response is an indication that the children will benefit from supplementation but that potential clinical outcomes are not yet measurable. Based on this very valid and relevant comment, we have altered our conclusion: It now reads as follows:

Lines 347 onwards: "In summary, our results show that daily vitamin B_{12} supplementation improves vitamin B_{12} status and the metabolic profile of Nepalese infants. The metabolic response indicate that this population could benefit from vitamin B_{12} supplementation, but the clinical consequences of subclinical deficiency in early childhood remain uncertain."

Related to this comment, we have also expanded the discussion to include a section on the metabolic pathways that were affected by vitamin B_{12} supplementation: lines 313-322 now reads: "Vitamin B_{12} is involved in two biochemical reactions in humans [31]. In one of these, vitamin B_{12} is required for the transfer of methyl groups, which includes remethylation of homocysteine to methionine. Disruption of this pathway increases homocysteine, affects gene regulation and DNA synthesis [31]. Vitamin B_{12} also acts as an enzymatic cofactor for methyl-malonyl CoA mutase, an enzyme involved in the catabolism of fats and amino acids. Disruption of the latter pathway explains the increased MMA observed in vitamin B_{12} deficiency. Our study provides causal evidence that both of these metabolic pathways are affected by mild vitamin B_{12} deficiency in children and their functioning improves with supplementation. Thus, despite no effects on the clinical outcomes, children in this population could benefit from increasing the vitamin B_{12} intake as indicated by their improved metabolic profile. "

- The authors rightfully discussed this topic in the manuscript, thus I would assume that they also consider the possibility that B12 supplementation can be beneficial for developmental markers, possible after dose adjustments. Thus, the current results may not be as exciting for the Bayley-III outcomes, but they are indicative of a selective metabolic response based on B12 status at baseline, which are not unexpected to be noticeable before notable changes in growth and development, and therefore important results for future studies in the field. I encourage to investigate the Bayley-III results between the B12-sufficient infants at baseline and the B12 deficient children to examine possible significant differences in the development tests (and not just exclude B12 sufficient children from the analysis), other than stunting. If the Bayley-III results do not differ by B12 status, then a non-response to the intervention is not surprising, since there wasn't a difference to begin with.

Response: Thank you for this comment. In our plan of analyses we predefined certain subgrouping variables, including B12 status. The sub-group specific effects are shown in supplementary figures 1 and 2. These variables, however, were not well described in the manuscript, in the updated manuscript we have therefore added the following sentence (Lines 218-221): "The subgrouping variables were stunting (defined as length for age z-scores <-2), underweight (weight for age z-scores <-2), low vitamin B_{12} status (3cB12<-0.5),

low birth weight (birth weight < 2,500 g), anemia (hemoglobin concentration < 11.0 g/dL), and exclusive breastfeeding."

- Moreover, the study included infants with sufficient B12 status, over 100 per group, which changes the sample size, and possibly effect size. We don't know if this may have affected the outcome, but it could be by reducing the sample numbers for certain statistical analyses the effect size just changed enough to not be significant. However, given the limited amount of studies on this topic, this manuscript does add to the knowledge base, but conclusions should be revisited.

Response: Thank you, please see the updated discussion both on external and internal validity and the consequence of including children with sufficient B12 status. We have also updated the conclusion.

- Line numbers are missing. Please add as per journal guidelines, which is also beneficial for the reviewer.

Response: Line numbers have now been added

- Please add a space before reference directly attached to the word, e.g. but not limited to P9 "charts[29]".

Response: Thank you, added throughout the manuscript.

Abstract:
- RDA is an abbreviation and should be explained at the first use.

Response: We have amended the text in the abstract as well as in the method section.

- As mentioned above, I do not recommend to generalize the results obtained to a population basis as some your results differ depending on the B12 status of the children.

Response: We have amended the text concerning generalizability of results within the Discussion.

- Author Summary: The meaning of the findings should be revised based on my discussion above.

Response: Agreed and amended as appropriate.

- Introduction: P4 last paragraph: A daily oral supplementation with 1.8μg?

Response: the word "oral" has been added.

- P4 last paragraph: I am not quite clear how the results from the 3 RCTs support that the public health concern is not sufficient to alter feeding recommendations, given

that all trials showed benefits of the B12 interventions. A little bit more about your thought process here would be beneficial to the reader.

Response: Thank you for the comment. We have clarified the text and changed to (lines 79-81 "The evidence from these RCTs support the notion that poor vitamin B_{12} status may be a relevant public health concern, however the evidence is currently not strong enough to alter widespread feeding recommendations.ons".

- Subjects and Methods: P6 Intervention and co-intervention: each sachet provided the daily dose of supplements.

Response: Agreed and amended.

- P7 1st line: home visits were conducted by whom?

Response: Please see lines 140 onwards which now include this information: "During weekly visits to the homes, field workers asked the mothers about intake..."

- P8 1st paragraph: please add model and manufacturer with location for the used scales and any equipment used for height measurements.

Response: We have used Seca portable weighing scales and length boards this information is now included in lines 166-169

- P8 first paragraph: please add reference(s) for the mentioned standard guidelines.

Response: Thank you, we have removed the term "standard guidelines" and updated the description of the anthropometric procedures to "Weight was measured with a portable electronic scale (model 877, Seca, California, USA) that measures to the nearest 0.01 kg, and length was measured with portable length board (model 417, Seca, California, USA). We measured length and weight at the clinic or home during the monthly follow up visits. All anthropometric measurements were performed twice. The mean values were used in the analyses."

- P8 1st paragraph: mean values of how many replicate measurements?

Response: Each measurement was performed twice. The lines 169-170 have been updated to include this information. Please also see the response to the previous question.

- P8 Laboratory procedures: HemoCue model?

Response: The hemoglobin concentration was analyzed using the HemoCue (HemoCue 201, Ångelholm, Sweden), now indicated within text (line 175).

- P8 Laboratory procedures: Temperature during centrifugation? Which model, manufacturer of the centrifuge?

Response: This information has now been included in lines 176-177: "The blood was centrifuged at room temperature for 10 min at 2000-2500 g within 10 min after venipuncture (Model R-304, Remi, Mumbai, India)."

- I assume samples were shipped on dry ice to Norway for analysis?

Response: Yes: and the following sentence has been added "The samples were shipped to Norway on dry ice by WorldCourier" now indicated in line 180.

- P8 Laboratory procedures: The B12 analysis is not based on the strain of bacteria but the strains were used for this.

Response: agree, «based on» replaced by "using"

- P8 Laboratory procedures: "The functional markers plasma homocysteine…". This sentence does not belong in the Methods section, but could be added in the Introduction and/or Discussion.

Response: Disagree, we believe the use of the term" functional markers" belongs in the methods section and throughout the manuscript, and have retained it as such.

- P9 statistics: Generally, looks good to me but I am not an expert in statistics.

Response: Thank you

- P9 statistics: You defined z-scores -2 based on the WHO growth charts? Please specify or add references.

Response: The reference (29) is provided in the analysis part of the method section. We have also added this reference to justify the cut-off at -2 z scores.

- P10: Results, 1st paragraph: 572 completed the Bayley-III, but did you also get blood from 572 children as well?

Response: Thank you for highlighting this issue. We had valid blood samples from 567 children. The text within the Results section (line 231) and figure 1 have been updated to indicate this.

- Results: P11, 2nd paragraph: "We did not find adverse effects…"

Response: changed to "We found no adverse effects of vitamin B_{12} supplementation (supplemental table 3)" (Line 266-267).

- Discussion: P11, Discussion: the 2nd sentence is unnecessary if you have to explain it in the third sentence. Please omit.

Response: Omitted

- Please add a Discussion about my points in the opening paragraph regarding your results and their interpretation.

Response: Thank you. Please see the updated discussion where we have included the points addressed previously. In the opening paragraph we clearly state that the supplementation resulted in improved vitamin B12 status in marginally stunted Nepalese infants and that those with poor status at the onset of the study benefitted the most. We did not however find any effect of the intervention on our main outcomes, which is also true when we restricted the analyses to those with poor status at baseline. In our understanding, this should now be clearly enough stated, and the points have been thoroughly addressed in the discussion in the updated manuscript.

- Tables and Figures: Where applicable, please add to all Tables footnotes and Figure legends the statistical test used to examine significant differences.

Response: Thank you, the footnotes have now been amended.

- Supplemental Table 3: 1st row = n (children), 2nd row = n (events). OK, but then the next 3 rows, which fall under n(events) appear to be n(children) undergoing the different numbers of events. The specific diagnosis, however, belongs with n(events). This needs to be clarified to the reader.

Response: Thank you, please see the amended supplemental table.

We have also made some minor edits to the text such as adding commons and punctuation throughout to improve the manuscript. We also renamed the "Communication" subscale of the Bayley Scales of infant development to "Language" which is more appropriate. In the first version of the manuscript we used both the term "blind" and "mask". In this updated manuscript we only use the term "blind".
